# Fine Optimization of Colloidal Photonic Crystal Structural Color for Physically Unclonable Multiplex Encryption and Anti‐Counterfeiting

**DOI:** 10.1002/advs.202305876

**Published:** 2024-04-04

**Authors:** Yifan Gao, Kongyu Ge, Zhen Zhang, Zhan Li, Shaowei Hu, Hongjun Ji, Mingyu Li, Huanhuan Feng

**Affiliations:** ^1^ Sauvage Laboratory for Smart Materials Shenzhen Key Laboratory of Flexible Printed Electronics Technology Harbin Institute of Technology (Shenzhen) Shenzhen 518000 China; ^2^ State Key Laboratory of Advanced Welding and Joining (Shenzhen) Harbin Institute of Technology (Shenzhen) Shenzhen 518000 China

**Keywords:** anti‐counterfeiting, encryption, fluorescence, inkjet printing, physically unclonable function, structural color

## Abstract

Robust anti‐counterfeiting techniques aim for easy identification while remaining difficult to forge, especially for high‐value items such as currency and passports. However, many existing anti‐counterfeiting techniques rely on deterministic processes, resulting in loopholes for duplication and counterfeiting. Therefore, achieving high‐level encryption and easy authentication through conventional anti‐counterfeiting techniques has remained a significant challenge. To address this, this work proposes a solution that combined fluorescence and structural colors, creating a physically unclonable multiplex encryption system (PUMES). In this study, the physicochemical properties of colloidal photonic inks are systematically adjusted to construct a comprehensive printing phase diagram, revealing the printable region. Furthermore, the brightness and color saturation of inkjet‐printed colloidal photonic crystal structural colors are optimized by controlling the substrate's hydrophobicity, printed droplet volume, and the addition of noble metals. Finally, fluorescence is incorporated to build PUMES, including macroscopic fluorescence and structural color patterns, as well as microscopic physically unclonable fluorescence patterns. The PUMES with intrinsic randomness and high encoding capacity are authenticated by a deep learning algorithm, which proved to be reliable and efficient under various observation conditions. This approach can provide easy identification and formidable resistance against counterfeiting, making it highly promising for the next‐generation anti‐counterfeiting of currency and passports.

## Graphical abstract

A robust anti‐counterfeiting solution is constructed by combining fluorescence and structural colors into a physically unclonable multiplex encryption system. By optimizing structural colors and integrating them with fluorescence, this work achieves the combination of secure encryption and convenient verification, which reconciles the conflict between high randomness encryption and easy authentication and promises advanced anti‐counterfeiting measures.

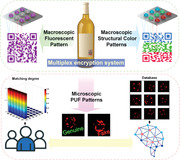

## 1 Introduction

The global problem of counterfeit products causes substantial economic losses and threats to individuals, enterprises, societies, and even national security.^[^ ^1^, ^2^, ^3^ ^]^ To address this problem, security labels employing various anti‐counterfeiting techniques have been commonly used to authenticate products and address issues of duplication and counterfeiting.^[^ ^4^ ^]^ For example, RMB banknotes have multiple security features such as watermarks, micro‐printing, holographic strips, transparent windows, intaglio printing, and special paper (Figure S1, Supporting Information), designed to provide legibility and security. However, achieving high security and identifiability using a single encryption technique remains a significant challenge.^[^ ^5^, ^6^ ^]^ The essential requirements for a practical security label include 1) excellent environmental durability, 2) simple manufacturing methods, 3) high complexity and encoding capacity to resist counterfeiting, and 4) readily recognizable encrypted information.

While several anti‐counterfeiting methods have been reported, a universal technique satisfying all of the above criteria has not been demonstrated. Photoluminescent phenomena, including fluorescence^[^ ^7^, ^8^ ^]^ and phosphorescence,^[^ ^9^ ^]^ along with nanostructure‐induced structural colors,^[^ ^10^, ^11^ ^]^ have been widely utilized in anti‐counterfeiting and encryption applications due to their tunable optical properties and facile functionalization features. Fluorescent patterns, invisible under natural light and visible under ultraviolet light (as observed on currency, Figure S1, Supporting Information), provide high concealment and security.^[^ ^12^, ^13^ ^]^ Various fluorescent patterns with ultra‐micro,^[^ ^14^ ^]^ full‐color,^[^ ^15^ ^]^ stimulus‐responsive,^[^ ^16^, ^17^ ^]^ or dual‐mode^[^ ^18^, ^19^ ^]^ attributes have been devised for encryption and anti‐counterfeiting. However, as chemical dyes, these fluorescent patterns may fade over time.^[^ ^20^, ^21^ ^]^ In contrast, structural colors based on interactions between geometrical nanostructure arrangement and light waves, such as refraction, diffuse reflection, diffraction, and interference, will not fade over time as long as the structure remains intact.^[^ ^22^, ^23^ ^]^ The color of the structural features can be adjusted over a wide range by changing the coloration methods (e.g., gratings,^[^ ^24^ ^]^ thin films,^[^ ^25^ ^]^ liquid crystals,^[^ ^26^, ^27^ ^]^ and photonic crystals^[^ ^28^, ^29^ ^]^) or component structures.^[^ ^30^, ^31^ ^]^ Benefiting from tunable photonic bandgap and easily customizable stimuli‐responsive properties, colloidal photonic crystals have become one of the most prevalent structural color materials in anti‐counterfeiting. For example, mechanical‐responsive,^[^ ^32^ ^]^ vapor‐responsive,^[^ ^33^ ^]^ and ion‐responsive^[^ ^34^ ^]^ colloidal photonic crystal materials have been widely developed. However, their practical application in encryption has been hindered by inherent limitations, such as low brightness and color saturation.^[^ ^35^, ^36^, ^37^ ^]^ In response, innovative approaches have been explored, including the use of melanin and SiO_2_ with a core‐shell structure, to enhance refractive index contrast and boost color saturation. Additionally, strategies combining the benefits of fluorescence and structural color for encryption and anti‐counterfeiting have been demonstrated, allowing versatile information encoding in both reflective and fluorescent modes.^[^ ^38^, ^39^ ^]^ However, these security labels can still be copied due to their deterministic fabrication processes, opening the possibility of duplication and counterfeiting.^[^ ^40^, ^41^ ^]^ To further enhance security and avoid deterministic fabrication processes, researchers utilized random processes such as random pinning, particle distribution, and amorphous structures to create physically unclonable security labels with fingerprint‐like features.^[^ ^42^, ^43^, ^44^ ^]^ These labels, complemented by deep learning techniques, offer robust artificial intelligence (AI) authentication. Therefore, integrating fluorescent and structured color security labels with a physical unclonable function (PUF) into products provides an excellent approach for achieving a multiplex encryption system, ensuring easy identification and formidable resistance against counterfeiting.

**Figure 1:**
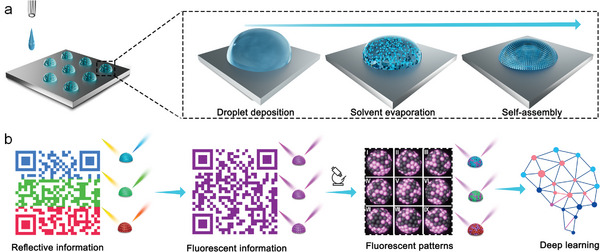
Schematic of inkjet printing of the evaporation‐induced self‐assembled colloidal photonic crystals and fluorescent/structural color multiplex encryption and anti‐counterfeiting: a) The colloidal photonic ink was printed on a hydrophobic, and as the solvent evaporated, the dispersed colloidal nanoparticles gradually approached each other, and one the concentration of colloidal nanoparticles reached a critical value, they started to crystallize and form hemispherical colloidal photonic crystal micro‐domes; b) colloidal photonic crystals exhibited colorful structural color patterns under natural light. The colloidal particles containing fluorescence at the same position showed distinct fluorescence patterns under UV light. Physically unclonable microscopic fluorescence patterns could be observed in specific regions using fluorescence microscopy, and deep learning was utilized to establish a database and verify authenticity.

In this study, a robust physically unclonable multiplex encryption system (PUMES) integrated with fluorescence and structural color was developed by programmable inkjet printing using colloidal photonic inks. The colloidal photonic inks were tailored for compatibility with inkjet printers, involving adjustments in the physicochemical properties, such as particle size, viscosity, and surface tension. The printing process on hydrophobic substrates facilitated the spontaneous assembly of colloidal nanoparticles into closely stacked hemispherical micro‐domes during solvent evaporation, optimizing the brightness and color saturation of the colloidal photonic crystal structural colors (**Figure** **1**a). Additional enhancements were achieved by tuning the printed droplet volume and addition with noble metals (Au), resulting in easily recognizable macroscopic structural color patterns. Subsequently, fluorescence was introduced, allowing for the generation of macroscopic dual‐mode patterns (fluorescence and structural color patterns) at the same position and microscopic PUF fluorescence patterns (Figure 1b). This dual‐mode encryption combining macroscopic fluorescence and structural color patterns provided strong identifiable encrypted information. Furthermore, evaporation process‐induced microscopic PUF fluorescence patterns exhibit uniform, unique, and uncorrelated random properties with high complexity and sufficient encoding capacity. These PUF patterns could be used to establish a database and authenticate security labels through deep learning.

PUMES utilizes facile evaporation‐induced self‐assembly and physically unclonable encryption to achieve a multiplex encryption system that is both easily recognizable and resistant to counterfeiting. While traditional encryption methods are based on fluorescence or structural colors regarding low environmental durability and low information concealment, respectively. PUMES, as a combined fluorescence and structural color encryption method, can circumvent these issues by integrating the advantages of both fluorescence and structural color methods. Moreover, PUMES avoids the deterministic fabrication process, which is commonly used in traditional encryption methods, preventing label replication and counterfeiting (Figure S2 and Table S1, Supporting Information). In comparison to traditional PUF methods, PUMES provide a breakthrough by providing accurate points for extracting encrypted information in a layered encryption system, ranging from macro to micro and from deterministic to indeterministic process (Figure S3 and Table S2, Supporting Information). This revolutionary combination of secure encryption and convenient verification reconciles the conflict between high randomness encryption and easy authentication, showcasing tremendous potential in anti‐counterfeiting for high‐value products.

## 2 Results and Discussion

### 2.1 Monodisperse Polystyrene for Colloidal Photonic Crystal Inks

**Figure 2:**
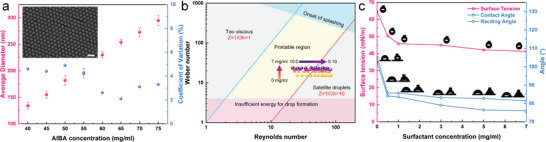
Colloidal photonic ink formulation from monodisperse polystyrene (PS) nanoparticles: a) Average particle size (red dots) and coefficient of variation (blue dots) of the PS nanoparticles at different initiator concentrations, where the inset shows an SEM image of the representative PS nanoparticles for nanoparticle size measurement (scale bar = 500 nm); b) phase diagram illustrating the printable/unprintable region of the colloidal photonic ink with different continuous phase compositions (from bottom to top, the surfactant concentration ranges from 0 to 7 mg mL^−1^; from left to right, glycol to formamide volume ratio ranging from 10:0 to 0:10); c) surface tension (red square dots), contact angle (blue diamond dots), and receding angle (blue round dots) of the colloidal photonic ink at different surfactant concentrations.

Achieving particle size monodispersity in synthesizing polystyrene (PS) was crucial, as it contributed to the spontaneous formation of highly ordered colloidal photonic crystal structures. Monodispersed PS were synthesized through emulsion polymerization,^[^ ^45^, ^46^ ^]^ exhibiting a spherical morphology, as observed in the scanning electron microscopy (SEM) images (**Figure** **2**a inset and Figure S4a–h, Supporting Information). The synthesis protocol is provided in the experimental section. The uniform particle size distribution, consistently below 5% coefficient of variation (standard deviation of particle size distribution/average particle size) under the same initiator concentration (blue dots in Figure 2a and Figure S4i, Supporting Information), met the requirement of polydispersity in forming colloidal photonic crystals. Additionally, the positively charged initiator imparted a zeta potential ranging from +20 to +40 mV to the PS nanoparticles (Figure S4j, Supporting Information). The substantial zeta potential generated electrostatic repulsion, which not only promoted colloidal stability by maintaining a specific particle distance but also aided in the spontaneous arrangement of nanoparticles into a highly ordered colloidal photonic crystal structure during the evaporation process.

Inkjet printing offers highly customizable pattern capabilities, making it an ideal tool for achieving patterned encryption and anti‐counterfeiting. However, it demands specific rheological properties of the ink, including appropriate viscosity and surface tension, to ensure a fluent printing process. Synthetic monodispersed PS nanoparticles dispersed in water with low viscosity and high surface tension (≈1 mPa·s and 70 mN m^−1^) would be unsuitable for inkjet printing. The viscosity and surface tension of the ink were adjusted by introducing a binary organic solvent (ethylene glycol and formamide) and surfactant (Brij‐35) in adjustable proportions to obtain suitable rheological properties of the ink.^[^ ^47^ ^]^ The surface tension and viscosity changes of the colloidal photonic inks with various continuous phase compositions are illustrated in Figure S5, Supporting Information. The formation of the ink droplets during inkjet printing typically consists of an intricate process. To better understand and describe the behavior of the ink droplets, several dimensionless physical parameters, including Reynolds (*Re*), Weber (*We*), and Ohnesorge (*Oh*) numbers, were employed. The mathematical expressions of these three are given by:

(1) $$Re=\frac{v\rho a}{\eta }$$

(2) $$We=\frac{v^{2}\rho a}{\gamma }$$

(3) $$Oh=\frac{\sqrt{We}}{Re}=\frac{\eta }{{\left(\gamma \rho a\right)}^{1/2}}$$

where *ρ*, *η*, and *γ* denote the density, dynamic viscosity, and surface tension of the ink, respectively, *ν* is the velocity of the ink droplet, and *α* is the characteristic length.

A phase diagram was constructed using *Re* and *We* as the X and Y coordinates, which provided an area for defining the appropriate composition of ink suitable for inkjet printing.^[^ ^48^ ^]^ The specific steps for constructing the phase diagram are illustrated in the Supporting Information and Figure S6, Supporting Information. The rheological parameters corresponding to each ink composition are plotted in the phase diagram (Figure 2b) when the nozzle diameter was set to 20 µm and the droplet speed was 7 m s^−1^.

Due to the high boiling points of ethylene glycol and formamide, this binary solvent exhibited a lower evaporation rate compared to pure water, which provided sufficient time for the PS nanoparticles to self‐assemble and form a closely stacked arrangement during evaporation. An optimal continuous phase composition of the colloidal photonic ink was selected with an ethylene glycol to formamide volume ratio of 9:1, considering the viscosity, surface tension, and evaporation rate. The corresponding viscosity and surface tension values of the ink under the optimal composition were 4.6 mPa·s and 65.2 mN m^−1^, respectively. The addition of a small amount of surfactant led to significant reductions in surface tension (red square dots in Figure 2c), contact angle (CA, blue diamond dots), and receding angle (RA, blue round dots) of the ink droplets. To ensure larger CA and RA values, the colloidal photonic ink was formulated without adding surfactants (star‐shaped dots in Figure S6a, Supporting Information). Notably, the stability of the inkjet process was closely associated with the waveform and voltage applied to the nozzle. Figure S6b, Supporting Information presents the customized voltage waveform designed for stable ejection of the colloidal photonic ink, indicating the flight of the ink droplets under this waveform. Mismatched rheological parameters with the waveform possibly resulted in satellite dots and trailing droplets (Figure S6c,d, Supporting Information). Utilizing multiple pulse waveforms ensured proper wetting of the nozzle and smooth detachment of the ink droplets during printing.

### 2.2 Modulating Photonic Crystal Structures via Diverse Substrate Hydrophobicities

For inkjet‐printed droplets, evaporation on the hydrophilic substrate led to the pinning of nanoparticles at the three‐phase contact lines (TCLs) and the formation of coffee rings, which impeded the self‐assembly process of the nanoparticles to form ordered hemispherical colloidal aggregates.^[^ ^49^, ^50^ ^]^ The reason was that the pined TCL would induce large cracks and vacancies in the colloidal aggregates. To address this issue, prior to printing, the substrates were treated with silane reagents to achieve a hydrophobic surface with freely sliding TCL.^[^ ^51^, ^52^ ^]^ The hydrophobic treatment process is illustrated in Figure S7, Supporting Information and described in the experimental section. By adjusting the ozone‐plasma treatment time (Figure S8a, Supporting Information) and silane treatment time (Figure S8b, Supporting Information), a hydrophobic substrate with large CAs and RAs could be obtained. This allowed the TCL to slide inward freely, facilitating the assembly of the colloidal nanoparticles and preventing the pinning and excessive aggregation of the nanoparticles at the TCL.

**Figure 3:**
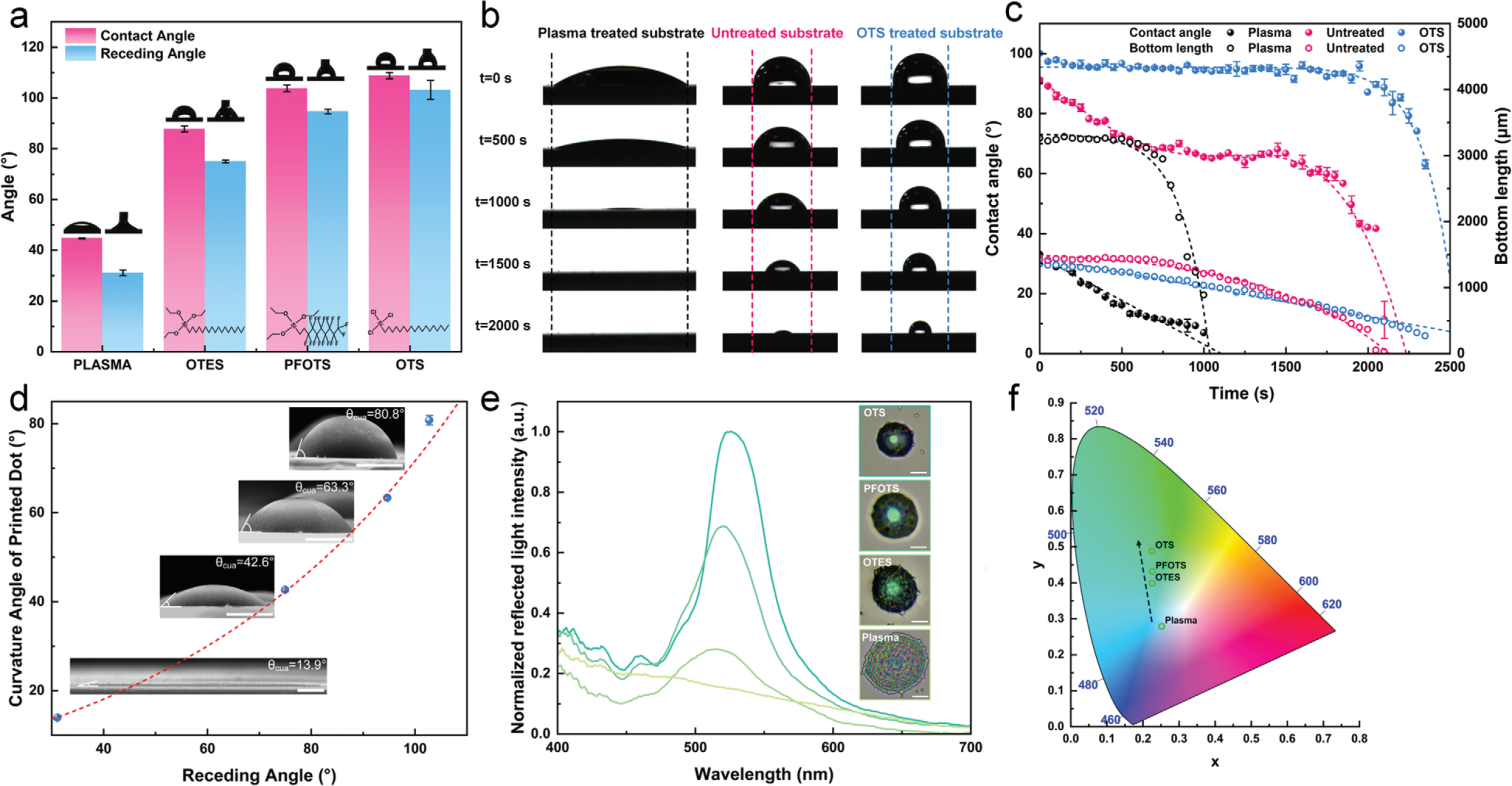
Modulating photonic crystal structures via diverse substrate hydrophobicities: a–c) Regulation of substrate properties: a) Contact angle and receding angle of the ink droplets on the substrate after plasma treatment and various silane treatments, from left to right: plasma, OTES, PFOTS, and OTS treatments; b) morphological changes of the droplets on the plasma‐treated (black), untreated (red), and OTS‐treated (blue) substrates during evaporation; c) variations in contact angle (solid dots) and bottom length (hollow dots) during droplet evaporation. d–f) Morphology of the colloidal photonic crystal and optical properties on different hydrophobic substrates: d) relationship between the receding angle of ink droplets on different hydrophobic substrates and the curvature angle of colloidal aggregates after drying (inset scale bar = 5 µm); e) reflectance spectra of the structural colors that formed on different hydrophobic substrates (inset scale bar = 5 µm); f) Commission Internationale de l'Éclairage diagram of the colloidal photonic crystals with different crystallinities.

The substrates were treated with various silane reagents to obtain substrates with varying degrees of hydrophobicity (**Figure** **3**a).^[^ ^53^ ^]^ The surface modifications included plasma treatment and sialylation with octadecyltriethoxysilane (OTES), 1H,1H,2H,2H‐perfluorodecyltriethoxysilane (PFOTS), and octadecyltrichlorosilane (OTS), respectively. The CA values of the treated substrates were 44.7° ± 0.2°, 87.8° ± 1.2°, 103.8° ± 1.3°, and 110.8° ± 1.2°, respectively, while the corresponding RA values were 31.1° ± 1.2°, 75.0° ± 0.5°, 94.7° ± 0.9°, and 102.8° ± 3.7°. The asymmetric stretching peak of Si‐O‐Si at 1112 cm^−1^ in the Fourier transform infrared spectrum confirmed the formation of chemical bonds between the self‐assembled monolayer (SAM) and the substrate (Figure S9a,b, Supporting Information). The significant increase in the intensity of the C 1s and F 1s peaks in the X‐ray photoelectron spectroscopy results further confirmed the presence of SAM on the substrate surface (Figure S9c,d, Supporting Information).

The ink droplets that evaporated on the substrates with different interfacial properties exhibited different TCL evolution. On the hydrophilic and untreated substrates, the TCL was pinned within the first 500 s of the evaporation process, leading to a rapid decrease in CA and thinning of the liquid layer at the edge. The evaporation rate at the TCL exceeded the rate at the center of the droplet, resulting in continuous outward capillary flow from the center to the edge, which replenished the liquid loss at the edge. Colloidal nanoparticles were carried to the edge of the droplet, leading to the formation of a coffee ring structure through stacking (black and red components in Figure 3b,c). On the OTS‐treated substrates (hydrophobic), the TCL of the droplet could freely slide, and the CA remained constant, fluctuating ≈100° for an extended period > 2000 s. This provided sufficient time for the colloidal nanoparticles to assemble into ordered crystal structures, thus, avoiding the coffee ring effect (blue parts in Figure 3b,c). Consequently, the sizes, morphology, and structures of the colloidal aggregates could be adjusted by modulating the interfacial properties of the printing substrate (Figure S10, Supporting Information). The ink droplets on the hydrophilic substrate (plasma treatment), which formed colloidal aggregates after solvent evaporation, were nearly flat with a very low height‐to‐diameter ratio (H/D = 0.047). However, as the hydrophobicity of the substrate increased, the crystallinity of the colloidal aggregates intensified, and the H/D ratio increased to 0.420. The corresponding 2D fast Fourier transform transitioned from bright concentric rings (indicative of short‐range ordered colloidal aggregates) to sharp hexagonal dot matrix patterns (indicative of long‐range ordered colloidal photonic crystals).

The photonic ink with an average particle size of 230 nm self‐assembled on the hydrophobic substrates, resulting in the formation of hemispherical colloidal photonic crystal micro‐domes. When illuminated with natural light, a distinct bright green spot was observed in the center of the micro‐domes. The reflection spectra exhibited a peak at ≈530 nm, which was attributed to the Bragg diffraction of the incident light by the periodically‐stacked colloidal nanoparticles. As the hydrophobicity of the substrate increased, the curvature of the colloidal photonic crystal micro‐domes (*θ_cua_*) also increased, leading to closely stacked colloidal particles with a higher reflection intensity (Figure 3d,e). By contrast, for the substrates treated with plasma (hydrophilic), the appearance of an integral white color was primarily attributed to incoherent light scattering by the short‐range ordered colloidal nanoparticles. As illustrated in the Commission Internationale de l'Éclairage (CIE) diagram, the coordinates of the structural colors that formed on different hydrophobic substrates (Figure 3f) gradually transitioned from (0.25, 0.28) (white, lower color saturation) for plasma treatment to (0.23, 0.49) (green, higher saturation) for OTS treatment. Therefore, hydrophobic substrate treatment allowed the attainment of structural colors with high brightness and color saturation.

### 2.3 Tailoring Print Droplet Volume for Enhanced Photonic Crystal Brightness

**Figure 4:**
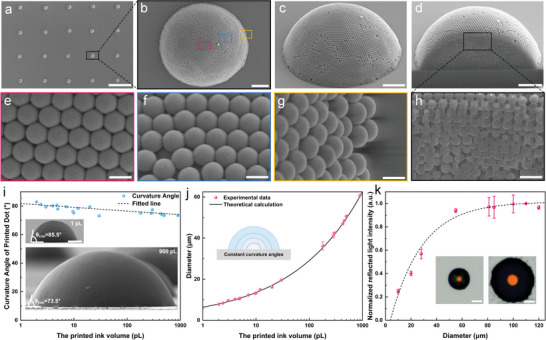
Tailoring print droplet volume for enhanced photonic crystal luminance: a) SEM images of the inkjet‐printed micro‐dome arrays (scale bar = 50 µm); b,c) top‐ and side‐views of the micro‐domes (scale bar = 2 µm); d) FIB cut out of a micro‐dome cross‐section of a colloidal photonic crystal (low magnification, scale bar = 2 µm); e–g) local enlargements of the top‐view of the micro‐dome at the top, sides, and bottom (scale bar = 200 nm); h) local enlargement of the cross‐section image of the FIB cutting micro‐dome (scale bar = 400 nm); i) curvature angle between the micro‐domes and the substrate formed by ink droplets with different volumes, where the insets show SEM images of the micro‐domes formed by the 1 and 900 pL ink droplets (scale bars = 1 and 10 µm, respectively); j) control of the micro‐dome diameter on the hydrophobic substrate by adjusting the ink volume; k) normalized reflected peak intensity of micro‐domes with different diameters, where the inset shows optical microscopy images of micro‐domes with different diameters captured under the same light intensity and aperture size (scale bar = 10 µm).

The use of programmable inkjet printing enabled precise control over the ink droplet volume, print pitch, and pattern design, facilitating the construction of large‐scale structural color micro‐dome arrays on hydrophobic substrates (OTS‐treated silicon wafers). The SEM images of the inkjet‐printed micro‐dome arrays revealed their nearly uniform size (**Figure** **4**a) and hemispherical shape (Figure 4b,c), with colloidal nanoparticles arranged in an ordered close‐stacked structure. Only a few defects were observed at the boundaries. Figure 4e–g presents the localized enlarged surface structures of the micro‐domes observed from the top (red), side (blue), and bottom (yellow) perspectives. These micro‐domes exhibited face‐centered cubic (FCC) structures with closely stacked colloidal particles. To further explore the inner structures of the micro‐domes, a focused ion beam (FIB) was used to obtain the cross‐section SEM images (Figure 4d,h). The internal portions of the micro‐domes did not show any hollow regions, and the colloidal nanoparticles were composed of closely stacked (111) faces of the FCC structure in the ordered layers.

The bright structural colors arose from constructive interference between the multiple layers of ordered stacked colloidal nanoparticles. However, due to the curved surface of the micro‐domes and the limited angular range of light that the objective lenses could collect, the photonic crystal micro‐domes showed bright and uniform colorful spots in the central region, while the edge regions were colorless (Figure S11a, Supporting Information). The objective lenses could collect reflected light from the central region of the micro‐dome (lattice parallel to the substrate). While light incident at larger angles interacted with the lattice and was not collected by the microscope objective, a geometric model was constructed to analyze the coherence between the collection angle of the actual objective lens and values calculated from the diameters of the spot and the micro‐domes (Figure S11b, Supporting Information). Further validation using objective lenses with different numerical apertures (NA) confirmed the correlation between the color region of the structural color and the angular range of the backward reflected light captured by the objective lenses. The objective lenses with higher magnification and larger NA were capable of capturing light reflected from a wider angular range, which increased the diameter of the observed colorful spot (Table S3, Supporting Information).

To further improve the brightness of the structured color, larger diameter micro‐domes were fabricated with a programmable inkjet printer by increasing the volume of the printed ink droplets. This was achieved by adjusting certain parameters, such as the drop time, voltage amplitude, and duty cycle, which influenced the ink droplet volume. Due to the uniform hydrophobicity of the substrate, the curvature angle of the micro‐domes (*θ_cua_*) remained essentially constant, with only a slight decrease from 85.5° to 73.5°, as the ink droplet volume increased from 1 to 900 pL (Figure 4i). For a fixed *θ_cua_*, a mathematical geometry model was established to regulate the volume of the printing ink so that the diameter of the micro‐domes could be precisely adjusted in the range of a few to tens or even hundreds of microns (Figure S12, Supporting Information). The micro‐dome diameter (*d_dome_*) and the volume of the printed ink droplet (*V_i_*) could be related as follows:

(4) $$d_{dome}=\sqrt[{3}]{V_{i}\times R\times A/\pi }$$

where R is the retention rate of the ink droplet after evaporation (≈0.05), and the product of the ink droplet volume and retention rate (*V_i_ × R*) will give the volume of the micro‐dome (*V_m_*), according to:

(5) $$A=\frac{24{\left(\sin\theta_{cua}\right)}^{3}}{\left(2+\cos\theta_{cua}\right){\left(1-\cos\theta_{cua}\right)}^{2}}$$

The diameter of the micro‐domes that formed after the ink droplet evaporated (red dot in Figure 4j) aligned with the theoretically calculated micro‐dome diameter (solid black line in Figure 4j). The representative SEM images illustrating this correspondence are shown in Figure S13a, Supporting Information.

The reflectance intensity and spectra (Figure 4d and Figure S13b, Supporting Information) illustrated that the intensity of the Bragg reflection peak increased with growing diameter of the micro‐domes, reaching a saturation value at a diameter of ≈50 µm. This phenomenon could be attributed to the influence of the internal structures of the colloidal photonic crystals on the reflection peak intensity. Cross‐sectional views of the micro‐domes prepared by FIB cutting (Figure S14, Supporting Information) revealed the hierarchical morphology of the colloidal photonic crystal. The surface region (Figure S14d, Supporting Information) consisted of several layers with closely ordered stacking colloidal particles in a curved 3D crystal structure. Toward the interior of the micro‐dome (Figure S14e, Supporting Information), the arrangement of colloidal particles gradually became disordered, and the thickness of the ordered layers on the surface progressively increased with increasing diameter of the micro‐domes (Figure S15, Supporting Information). Additionally, numerical simulations were conducted by constructing models representing colloidal photonic crystals with varying numbers of ordered layers, which were consistent with the experimental results (Figure S16, Supporting Information). Therefore, the observed increase in reflection intensity with growing micro‐dome diameter could be explained by the progressive increase in the thickness of ordered layers on the surface. Increasing the diameter of the colloidal photonic crystal micro‐domes provided a higher degree of crystallization, which augmented the peak reflection intensity of structural color. Nevertheless, once the thickness of the ordered layer reached a certain value, the reflection of the incident light reached saturation, and the reflection intensity no longer increased with a further increase in the thickness of the ordered layer.

### 2.4 Optimizing the Color Saturation of Multi‐Chromatic Structural Colors

**Figure 5:**
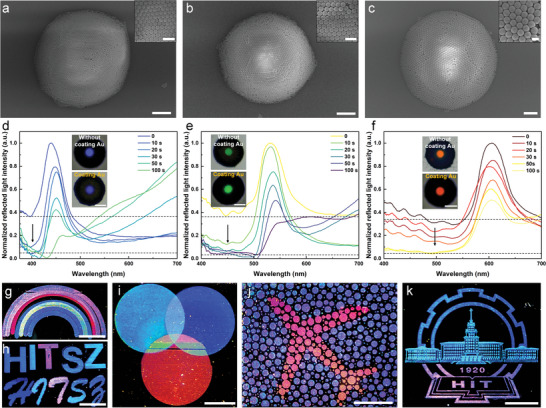
Optimizing Color Saturation of Multi‐chromatic Structural Colors: a–c) Top‐view SEM images of the colloidal photonic crystals consisting of PS nanoparticles with particle sizes of 180, 230, and 254 nm, respectively (scale bar = 2 µm), where the inset shows a magnified view of the central part of the micro‐domes, revealing hexagonal close stacking of the PS nanoparticles (scale bar = 500 nm); d–f) influence of addition selective spectral absorbers (Au nanoparticles) on the reflection spectra of structural colors, where the inset compares the structural color under an optical microscope before and after addition with Au nanoparticles (scale bar = 10 µm); g–k) inkjet‐printed patterns showcasing the colorful structural colors of the colloidal photonic crystals.

The coloration of structured color could be tuned throughout the visible spectrum by changing the diameter of the colloidal particles. Multi‐chromatic structural colors were generated using colloidal nanoparticles with average particle diameters of 180, 230, and 254 nm (**Figure** **5**a–c), resulting in Bragg diffraction peaks located at 437, 532, and 606 nm (the blue, green, and red solid lines in Figure S17a, Supporting Information), respectively. Optical microscopy images demonstrated the vivid blue, green, and red structural colors of these micro‐domes (inset in Figure S17a, Supporting Information). The color coordinates of the corresponding reflectance spectra in the CIE diagram were (0.15, 0.06), (0.23, 0.49), and (0.44, 0.31), respectively (Figure S17b, Supporting Information). Additionally, the measured reflection spectra by the fiber‐optic spectrometer coincided with the results calculated by the Bragg‐Sneer law (Table S4, Supporting Information). The reflection peak wavelength was found to be related to the diameter of the colloidal nanoparticles composing the photonic crystal. To validate this conclusion, a theoretical model based on the FCC structure with colloidal particles aligned in the X‐Y plane along the (111) surface was employed, as detailed in the experimental section. The reflection spectra obtained from the simulations (dashed line in Figure S17a, Supporting Information) exhibited remarkable agreement with the experimentally measured spectra, revealing direct proportionality between the peak wavelength of the structural color and the particle diameter of the colloidal nanoparticles (Figure S17c, Supporting Information).

The inherent constraints of the hemisphere micro‐domes led to internally disordered structures, resulting in broadband scattering and a whitish macroscopic appearance. Traditional approaches have involved the addition of melanin, such as polydopamine, a material with broadband absorption, to suppress scattering and enhance the contrast between the spectral background and Bragg diffraction peak.^[^ ^31^, ^54^, ^55^, ^56^ ^]^ However, these melanin materials can exhibit non‐selective absorption properties, resulting in a decrease in the intensity of the Bragg diffraction peak responsible for generating structural color. In this work, the localized surface plasmon resonance (LSPR) effect between the metal nanoparticles and electromagnetic waves (typically in the form of light) was leveraged to induce the collective oscillation of free electrons on the nanoparticle surfaces, resulting in selective light absorption at the resonant frequency. The resonant frequency depended on the size, shape, and composition of the nanoparticles.^[^ ^37^ ^]^ For example, noble metal nanoparticles such as gold (Au) and silver (Ag) can easily tune their absorption peaks within the visible spectrum region (Figure S18a, Supporting Information, simulated LSPR absorption peaks for 20 nm Au and Ag nanoparticles centered at 521 and 420 nm, respectively). These nanoparticles can serve as spectrally selective absorbers, selectively absorbing incoherent scattering from disordered structures.^[^ ^57^, ^58^ ^]^ In this work, the tunable absorption peaks minimized the attenuation of the photonic crystal Bragg diffraction peaks, enhancing the color saturation of structural colors with minimal impact on their brightness.

Au nanoparticles were used as optimized selective absorbers for enhancing color saturation (experimental section). This was attributed to the more suitable absorption range of the Au nanoparticles (400–550 nm) compared to the Ag nanoparticles (400–450 nm), effectively absorbing scattering generated by the disordered colloidal nanoparticles. Additionally, Au nanoparticles were more stable and less susceptible to environmental influence, and the simulated absorption peaks remained constant with varying array sizes (Figure S18b, Supporting Information). The changes in reflected spectra before and after sputtering Au nanoparticles (Figure 5d–f) combined two spectral features. Specifically, selective absorption induced by plasmon resonance reduced the scattered light intensity in the lower‐wavelength part of the spectrum without affecting the higher‐wavelength region with Bragg diffraction peaks. By selectively suppressing lower‐wavelength scattering light through the sputtering of Au nanoparticles on the surface of ordered‐layer colloidal photonic crystals, the contrast was enhanced without significantly affecting the brightness, resulting in macroscopically vivid colors.

To further validate this conclusion, a hierarchical model was established with Au nanoparticles (particle size of 20 nm), ordered PS colloidal arrays (particle size of 260 nm), and disordered PS nanoparticles. The introduction of disordered PS nanoparticles generated scattering light that matched the measured reflected spectra. The simulation results showed that adding Au nanoparticles to the ordered layer of colloidal nanoparticles significantly reduced the spectral reflectance baseline and minimized interference with the main Bragg diffraction peak (Figure S19a, Supporting Information). The color saturation of the structural colors increases within a certain range, but then decreases slightly when Au nanoparticles are excessive addition (Figure S19b, Supporting Information). This could be attributed to the reduction in gap distance between the Au nanoparticles or a gradual increase in their particle diameter with increased sputtering time, which resulted in a redshift in the absorption spectrum (Figure S20, Supporting Information). The changes in the color coordinates in the CIE diagram (Figure S21, Supporting Information) also indicated that adding Au nanoparticles increased the color saturation of the green and red structural colors. Further color saturation optimization of the structural colors in the colloidal photonic crystals could be achieved by adjusting parameters such as the shape of metal nanoparticles (e.g., square or triangular) and their deposition positions (e.g., disordered layers) in future studies.

In the structural color printing strategy, the optimization involved tuning the hydrophobicity of the substrate, adjusting the volume of the ink droplets, and incorporating selective spectral absorbers (Au) to achieve bright and vivid color patterns. By utilizing colloidal nanoparticles with different particle sizes, multi‐chromatic structural color patterns, such as a rainbow, regular and italic letters, tricolor plates, color blindness test diagram, and our institution logo (Figure 5g–k) were achieved using a programmable inkjet printer. When illuminated with natural light, these customizable patterns were easily distinguishable with the naked eye, thus, establishing a solid foundation for their potential application as macroscopic security labels.

### 2.5 Physically Unclonable Multiplex Encryption System

**Figure 6:**
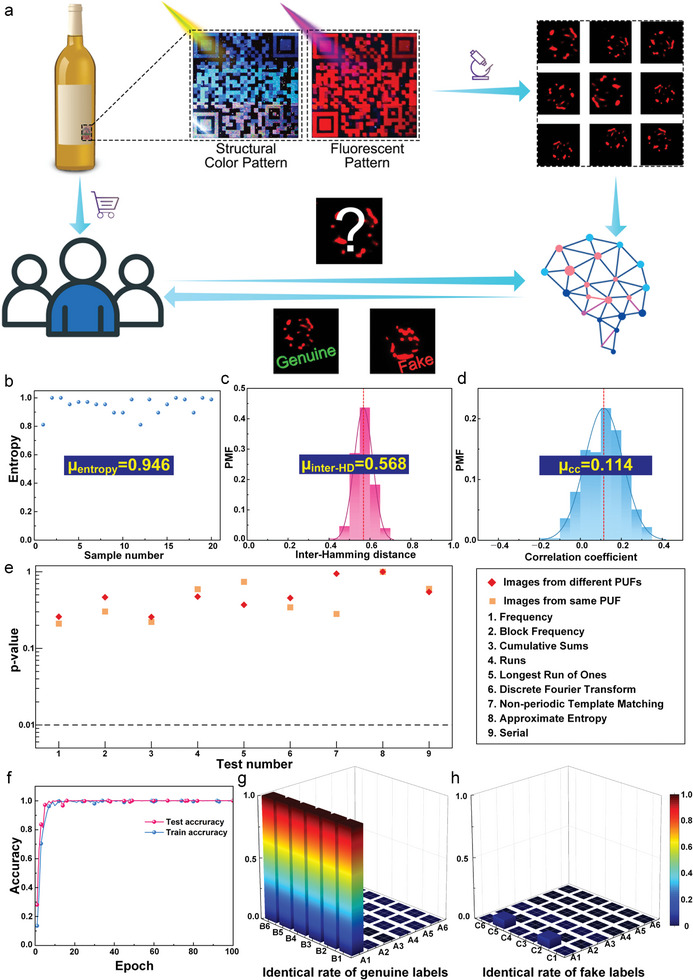
Physically unclonable fluorescence and structural color multiplex encryption system: a) Schematic of the multiplex encryption and authentication process, incorporating macroscopic structural colors, fluorescent patterns, and microscopic physically unclonable fluorescent patterns; b–d) randomness evaluation using statistical parameters (entropy, inter‐hamming distance, and correlation coefficient); e) randomness evaluation using the National Institute of Standards and Technology test; f) accuracy graphs for training (blue) and authentication (red) when training the datasets of security labels; f,g) matching degree of the deep learning model for labels c1–c6 and d1–d6, with c1–c6 corresponding to patterns derived from b1 (genuine product), and d1–d6 corresponding to labels not present in the database (fake product); color scale, ranging from blue to red, representing the matching degree (0 to 1) of the input images with the labels.

Upon obtaining macroscopic structural color patterns, the encryption level was further improved by introducing fluorescent nanoparticles with similar properties (particle size and Zeta potential) to the blank nanoparticles (Figure S22, Supporting Information). The introduction of fluorescence allowed for advantageous complementarity with structural color, thus, constructing a multiplex encryption system, as detailed in the Supporting Information and Figure S23, Supporting Information. The macroscopic structural color and fluorescent patterns provided readily identifiable dual‐mode encryption, while the microscopic random fluorescence patterns in the encryption system had physically unclonable properties, making counterfeiting difficult. The design and authentication process of the PUMES is shown in **Figure** **6**a. The resultant PUMES, exemplified by its application on a wine bottle, exhibited macroscopic structural color patterns and fluorescent patterns co‐existing in the same position under natural light and UV light, respectively. The macroscopic security labels enabled dual‐mode encryption in the visible and UV wavelength ranges. Furthermore, when observed under fluorescence microscopy, physically unclonable microscopic fluorescent patterns could be observed in a specific region due to the random distribution of fluorescent and blank particles during evaporation. These random fluorescence patterns served as PUF security labels, forming a database for training an AI system through deep learning.^[^ ^59^, ^60^, ^61^ ^]^ The PUF security label was created through a simple evaporation process. Results from the randomness test, as depicted in Figure S24, Supporting Information and Figure 6b–e, demonstrated that the PUF security labels exhibited uniform, unique, and uncorrelated characteristics, enhancing their high complexity. Moreover, their intricate fluorescence patterns provide sufficient encoding capability (Figure S25, Supporting Information).^[^ ^62^ ^]^ In contrast to low‐complexity PUF security labels, which might be recreated using forceful methods, those created using this approach exhibited high complexity and high encoding capacity, making them extremely difficult to reproduce or copy.

Figures S26 and S27, Supporting Information illustrate the database generation of PUF security labels. Six representative fluorescent patterns (Figure S26a, Supporting Information) were captured from specific positions to establish a PUF security label database. Each image (e.g., a1) was subjected to rotation, enlargement, reduction, and random positioning, providing 500 images for the AI system to learn and classify, as detailed in the experimental section. The representative images are shown in Figure S27, Supporting Information. A total of 3000 images were used to establish the deep learning database, and these images were divided into two groups, with 80% used for learning and 20% for authentication. After each learning cycle, the images designated for authentication were tested by the AI engine, producing a training accuracy graph, and this process was repeated until satisfactory recognition accuracy was achieved. After ≈10 epochs, the recognition accuracy stabilized at 1 (Figure 6f).

For the consumer authentication process, the macroscopic patterns serve as the initial authentication feature of the security label. As a result, consumers could easily discern macroscopic structural colors and fluorescent patterns under natural and UV light, respectively, with the naked eye. Access to the encrypted information is made possible using a smartphone. If consumers require further confirmation, PUF security labels containing microscopic fluorescent patterns can be obtained with a fluorescent microscope. The acquired pattern could then be uploaded to an AI system for comparison with the database of genuine products, and the corresponding deep learning engine would promptly provide the authentication results (genuine or fake) for consumers. Fluorescent images were captured with various magnifications, resolutions, brightness levels, rotation angles, and combinations of these factors (Figure S26b, Supporting Information) to mimic the consumer authentication process. These images encompassed all possible variations in imaging equipment, imaging conditions, user habits, and other factors that could potentially occur under actual authentication scenarios, and the images were input into the AI system for authentication. Although the AI had not previously learned these images (b1–b6), they originated from the same genuine product as (a1) in the database, resulting in a matching degree of ≈0.999 (Figure 6g). For comparison, six fluorescent patterns not exist in the database (Figure S26c, Supporting Information) were presented to the deep learning engine using the same authentication process. The results consistently indicated that the matching degree of the fake labels was below 0.5 (Figure 6h). Consequently, the matching threshold for distinguishing between genuine and fake security labels was set at 0.5. This allowed the deep learning engine to rapidly compare the matching degree of the security labels with the set threshold (genuine: matching degree ≥ 0.5, fake: matching degree < 0.5) and promptly provide authentication results to the consumer within seconds. To demonstrate the versatility of this model, five additional PUF fluorescent patterns (a2–a6) were also authenticated in the same manner, using a matching threshold of 0.5 to differentiate between the genuine and false labels, resulting in a false alarm rate of 0 (Figure S28, Supporting Information).

## 3 Conclusions

We demonstrated a physically unclonable multiplex encryption system (PUMES) integrated with fluorescence and structural color by programmable inkjet printing monodisperse colloidal photonic inks. Stable colloidal photonic inks compatible with inkjet printers were obtained by fine‐tuning the physicochemical properties, including the particle size, viscosity, and surface tension. Bright and vivid structural colors were achieved by improving the crystallinity of the colloidal photonic crystals and selective absorption of incoherent scattering through external optimization (hydrophobicity of the substrate), internal optimization (printed droplet volume), and the localized surface plasmon resonance effect of noble metals nanoparticles (Au), respectively.

Furthermore, we constructed robust PUMES that reconciled the trade‐off between high complexity encryption and convenient authentication. The macroscopic fluorescent and structural color patterns provided different information under natural and UV light conditions, which facilitated legibility with sufficient encoding capacity. The physically unclonable fluorescent patterns with sufficient randomness and encoding capacity were employed to establish a database and verify the authenticity using deep learning, enabling rapid (≈2 s) and accurate (0 false alarm rate) authentication.

This system provides a promising approach for combating counterfeiting and facilitating the practical application of fluorescent and structural colors for anti‐counterfeiting of high value products.

## 4 Experimental Section

### Materials

Chemical reagents consisting of anhydrous ethanol, cyclohexane, tetrahydrofuran, ethylene glycol, and formamide were obtained from Baishi Chemical Industry Co., Ltd. (Tianjin, China), while octadecyltrichlorosilane (OTS), 1H,1H,2H,2H‐perfluorodecyltriethoxysilane (PFOTS), and octadecyltriethoxysilane (OTES) were obtained from Macklin Biochemical Technology. Co, Ltd. (Shanghai, China). In addition, styrene, polyvinylpyrrolidone (PVP, Mw≈58 000), and 2,2′‐azobis[2‐methylpropionamidine] dihydrochloride (AIBA) were obtained from Merck Chemical Technology Co., Ltd. (Shanghai, China). All reagents were of analytical grade and used without any further purification. Ultrapure water was prepared by a Milli‐Q water purification system (resistance >18 MΩ cm). Silicon wafers were purchased from Lijing Technology Co., Ltd. (Zhejiang, China).

### Characterization

The morphology of the synthesized monodisperse PS nanoparticles and colloidal photonic crystal micro‐domes was characterized by field emission scanning electron microscopy (Gemini 560, Zeiss, Germany). The fluorescence spectra were measured using a fluorescence spectrophotometer (RF‐6000) with a fixed slit width of 5 nm. The surface potentials of the synthesized nanoparticles were measured by the nanoparticle size and surface potentiometers (Zetasizer Nano ZSP, Malvern, UK). The viscosity and surface tension of the colloidal photonic ink were measured with a digital viscometer (NDJ‐9S, Fangrui, China) and a surface tension meter (K‐100, KRUSS, Germany) under atmospheric conditions, respectively. The composition of the SAM on the silane‐treated substrates was characterized using an FTIR absorption spectrometer with an ATR attachment (Nicolet iS50, Thermo Fisher, USA) and an X‐ray photoelectron spectrometer (Versaprobe 4, ULVAC‐PHI, Japan). Optical images of the colloidal photonic crystals were captured using reflectance‐type optical microscopy (DM4 M, Leica, Germany) in bright field mode with illumination from a 50 W LED light source. The microregion reflectance spectra were obtained using a combined microscope (iM1, Olympus, Japan) and a fiber optic spectrometer (NOVA, Ideaoptics, China) with illumination from a 50 W halogen lamp and 50× objective lens (NA = 0.75). A clean silicon wafer served as the reflectance standard, and a sponge provided diffuse background light. The cross‐sectional morphology of the colloidal photonic crystals was observed using a dual‐beam scanning electron microscope (Crossbeam 350, Zeiss, Germany). The ultra‐micro fluorescent patterns were observed using a fluorescence microscope (Axioscope5, Zeiss, Germany).

### Synthetic Monodispersed PS Nanoparticles

Monodispersed PS particles were synthesized through emulsion polymerization of styrene monomers in an aqueous polyvinylpyrrolidone solution (PVP) using the hydrophilic initiator 2,2′‐azobis[2‐methylpropionamidine] dihydrochloride (AIBA). The PS nanoparticle size (range from ≈100 to 300 nm) could be controlled by adjusting the initiator concentration (45 to 75 mg mL^−1^). Prior to the reaction, the styrene monomer was repeatedly washed three times in a solid phase extraction column containing alkaline alumina to eliminate trace amounts of polymerization inhibitors. In the reaction, 44 mL of ultrapure water, 5.5 mL of styrene, and 5 mL of 76 mg mL^−1^ of PVP solution were combined in a three‐necked flask. The flask, equipped with a condenser and connected to a nitrogen gas source, was securely positioned on an oil‐heated bath. A magnetic stirrer mixed the contents at 800 rpm for 30 min, and nitrogen gas purged the residual oxygen. Subsequently, 1 mL of an aqueous AIBA initiator solution was added, and the flask was warmed to 70 °C under nitrogen protection for 8 h. Afterward, the reacted emulsion was subjected to centrifugation at 11 000 rpm for 15 min, followed by washing with deionized water three times.

### Preparing the Colloidal Photonic Ink

The PS nanoparticles dispersed in deionized water (10 wt%) were combined with a binary solvent (ethylene glycol and formamide) at an adjustable proportion to formulate a colloidal photonic ink that was compatible with inkjet printing. The volume ratio between the binary solvent to the PS nanoparticles dispersion was set at 7:3. For the binary solvent, the optimal ratio of ethylene glycol to formamide was 9:1. To obtain large CA and RA values of the ink droplets, the addition of surfactant (Brij‐35) was omitted to avoid any reduction in surface tension.

### Fabrication of Substrate Hydrophobicity and Measurement of Contact Angle

Three silane reagents (OTS, OTES, and PFOTS) were used to enhance the hydrophobicity of the silicon wafer. Silicon substrates were initially sonicated in acetone, anhydrous ethanol, and deionized water for 5 min to eliminate contaminants on the silicon surface, followed by nitrogen gas drying. The substrates were then treated with an ozone‐plasma clean device (TS‐1008, Tonson, China) at 100 W for 300 s to introduce hydroxyl groups, thus, rendering the silicon surface sufficiently hydrophilic. Subsequently, the hydroxyl‐modified substrates were immersed in silane solutions (with cyclohexane or toluene as diluents, in a silane‐to‐diluent volume ratio of 1:99) at room temperature overnight to obtain silicon substrates with varying hydrophobicity. Finally, the treated substrates were sonicated in tetrahydrofuran for 15 min to remove all unbound surface silane. The substrates were then rinsed with ethanol, deionized water, and wiped with dust‐free paper to eliminate any residual organic reagents and maintain a shiny surface for inkjet printing.

The CAs were measured using a contact angle meter (LSA100, Lauda, Germany). First, a 1 µL droplet was gradually placed onto the substrate surface using a micro syringe, allowing it to transition smoothly due to surface tension. ImageJ software was used to analyze the captured static contact patterns and determine the CA. To characterize the evolution of the TCL of the ink droplets, a 0.5 µL of ink was extracted from the droplet using a micro syringe, and the maximum CA, when the TCL shrank, was measured as the RA. Additionally, photographs of the droplets were captured every 10 s to observe the dynamic change in CA during evaporation.

### Patterning

The structured color patterns, composed of arrays with colloidal photonic crystal micro‐domes, were fabricated using either a high‐resolution material deposition instrument (DMP 2850, Fujifilm Dimatix, Japan) or an electrofluidic inkjet printer (EHDJet‐H, Sygole, China). ≈1.5 mL of the colloidal photonic ink was loaded into the cartridge using a syringe. Then, the waveform and voltage were adjusted to ensure smooth ejection, preventing the formation of long trails or satellite dots and ensuring a flawless structure of the evaporated photonic crystals. The flight of the ink droplets was observed by the attached droplet observation device (shutter speed = 1/1000 s). The nozzle height and starting point were adjusted based on the substrate thickness and position. The nozzle was cleaned every 30 lines to prevent nozzle clogging during printing. The ink was printed and evaporated at room temperature.

### Numerical Simulation of Reflectance and Absorption Spectra

The theoretical reflection and absorption spectra were computed using the commercial finite element software, FDTD (Lumerical Solutions, https://www.lumerical.com/tcad‐products/fdtd). An FCC colloidal lattice with a (111) orientation in the X‐Y plane was employed to model the ordered layers, while randomly distributed particles simulated the disordered layers in the colloidal photonic crystals. Refractive indices were consistent with the actual values for PS (*n* = 1.587). A plane wave light source with a wavelength range of 400–700 nm was set incident vertically downward onto the (111) face. The reflection spectra were obtained using a frequency domain field profile monitor above the light source with 100 frequency points to ensure smooth spectra. A non‐auto‐uniform grid type with an accuracy of 2 and grid size of ≈10 nm, significantly smaller than the particle size (150–300 nm), was chosen to expedite calculations. Perfectly matched layers served as the boundary conditions in all three directions (XYZ) to prevent interference phenomena at the periodic boundary conditions and ensure convergence.

For the numerical simulations of the gold nanoparticle absorption spectra, an array of gold nanoparticles was constructed by varying the array size (from 5 × 5 to 50 × 50), particle diameter (from 10 to 100 nm), and pitch (from 10 to 100 nm). The absorption spectra were obtained using a monitor below the array. The refractive index of the gold nanoparticles was set to 0.47, with the remaining settings consistent with the calculation of the reflection spectra.

### LSPR for Optimizing Structural Color

Gold nanoparticles (Au) were used to enhance the color saturation of the colloidal photonic crystals. After the ink droplets dried and formed colloidal photonic micro‐domes, they were sputtered by an ion sputterer (JS‐18064, KYKY, China) to deposit Au on the surface of the micro‐domes using Au targets. The deposition amount was adjusted by controlling the sputtering time.

### Deep Learning and Validation Methodology

Deep learning was conducted using the AlexNet convolutional neural network, with the code executed in Pycharm software, and all deep learning networks were based on the PyTorch framework. To ensure an adequate training set for the AlexNet model, a data augmentation procedure was applied to the original images. First, a fluorescent pattern image captured by a microscope was adjusted to 512 × 512 pixels based on the pixel‐region relations. Subsequently, an image algorithm identified and adjusted the pattern fill density to 0.8 if it was below the threshold. Finally, the fluorescent pattern was rotated in steps of 0.72° for 360°, with random placements during rotation, generating 500 images for training (80%) and validation (20%). Following 10 learning cycles, the validation accuracy fluctuated ≈100%.

In practical applications, when a consumer submits a randomly captured photo of a genuine security label to the AI using fluorescent microscopy, the system automatically retrieves exact correspondence information and provides a detailed matching degree with the index name. For images not previously learned, such as those from counterfeit goods, the AI assigned a lower matching degree. Deep learning operating as a black box would allow consumers to verify the authenticity of goods containing PUF security labels without requiring an understanding of the underlying mechanisms.

## Conflict of Interest

The authors declare no conflict of interest.

## Supporting information

Supporting Information

## Data Availability

The data that support the findings of this study are available from the corresponding author upon reasonable request.
