# Supplementary material for: Fine Optimization of Colloidal Photonic Crystal Structural Color for Physically Unclonable Multiplex Encryption and Anti‐Counterfeiting
Source: Adv Sci (Weinh). 2024 Apr 4;11(20):2305876. doi: 10.1002/advs.202305876 (PMC11132029; doi:10.1002/advs.202305876)
Supplement: Supplementary file 1 — Supporting Information [file ADVS-11-2305876-s001.pdf]

ADVANCED SCIENCE
Open Access

Supporting Information

for *Adv. Sci.*, DOI 10.1002/advs.202305876

Fine Optimization of Colloidal Photonic Crystal Structural Color for Physically Unclonable Multiplex Encryption and Anti-Counterfeiting

*Yifan Gao, Kongyu Ge, Zhen Zhang, Zhan Li, Shaowei Hu, Hongjun Ji, Mingyu Li and Huanhuan Feng**

Supporting Information

Fine Optimization of Colloidal Photonic Crystal Structural Color for Physically Unclonable Multiplex Encryption and Anti-Counterfeiting

*Yifan Gao[1], Kongyu Ge[1], Zhen Zhang[1], Zhan Li[1], Shaowei Hu[2], Hongjun Ji[2], Mingyu Li[2] and Huanhuan Feng[1]**

1 Sauvage Laboratory for Smart Materials, Shenzhen Key Laboratory of Flexible Printed Electronics Technology, Harbin Institute of Technology (Shenzhen),
Shenzhen 518000 (China)
E-mail: fenghuanhuan@hit.edu.cn

2 State Key Laboratory of Advanced Welding and Joining (Shenzhen), Harbin Institute of Technology (Shenzhen)
Shenzhen 518000 (China)

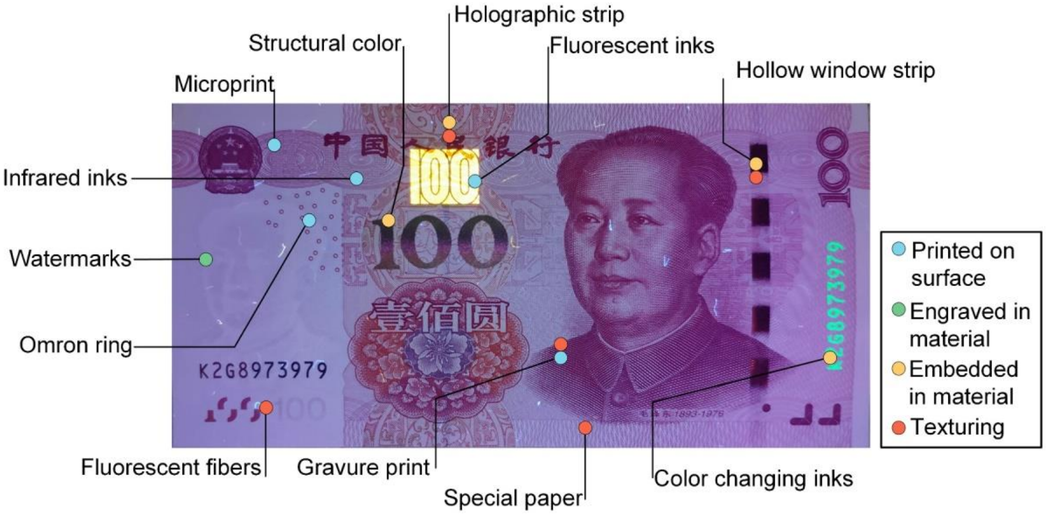

Figure S1. Various anti-counterfeiting methods are used in an RMB banknote. These anti-counterfeiting techniques can be classified according to their integration into the RMB: printed on the surface (blue), engraved in the material (green), embedded in the material (yellow), and texturized (red).

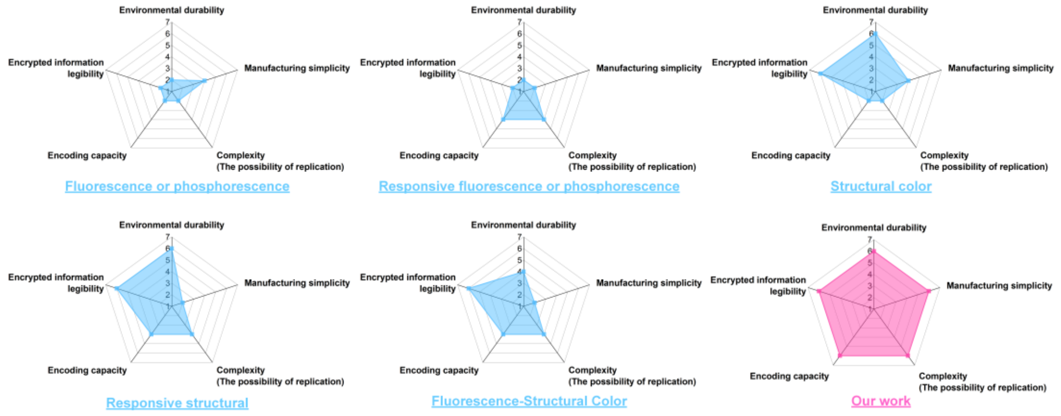

Figure S2. Radar plots evaluate critical requirements of the state-of-the-art anti-counterfeiting techniques. The marks are only general qualitative indications, with a higher score indicating favorable performance.

We generated radar charts comparing key requirements of anti-counterfeiting technologies to demonstrate the superiority of our fluorescence and structural color-integrated physical unclonable multiplex encryption system over traditional anti-counterfeiting techniques. The parameters described in Figure S2 and Table S1 were selected according to critical criteria necessary for the practical application of anti-counterfeiting techniques. Evaluation criteria included environmental durability, manufacturing simplicity, complexity (possibility of replication), encoding capacity, and legibility. The qualitative scoring assessment criteria and justifications are as follows:

Environmental durability:

In practical applications, optical security labels require excellent environmental durability to ensure the normal extraction of encrypted information with the passage of time or drastic changes in the environment. However, security labels based on fluorescence (or phosphorescence) involve chemical dyes, so these patterns may fade over time. Therefore, the fluorescence (or phosphorescence) and responsive fluorescence (or phosphorescence) methods are given scores of 2 in environmental durability. Structural color originates from the interaction between nanostructures and light waves, and it does not fade over time as long as the structure remains intact. The structural color and responsive structural color methods receive scores of 6. For the fluorescence-structural color method, the fluorescent component may partially lose

encrypted information due to environmental influences, resulting in a score of 4 for this method. In our work, the colloidal photonic crystal structure encapsulates the fluorescent dye, ensuring good environmental durability while achieving the combination of fluorescence and structural color, scoring of 6.

Manufacturing simplicity:

The simplicity of production of security labels directly influences their cost in practical applications. Security labels produced through a single technique have a very simple manufacturing process. Hence, the scores for fluorescence (or phosphorescence) and structural color methods are 6. Responsive security labels, requiring the incorporation of substances responsive to external stimuli, increase the manufacturing complexities, scoring 4 for responsive methods. The fluorescence-structural color integrated method needs to consider the compatibility of two distinct properties, resulting in a score of 2. In our work, the characteristics of fluorescent nanoparticles align with blank nanoparticles, remaining unaffected during final structure formation. A simple evaporation process yielded self-assembled photonic crystal structures and microscopic physically unclonable patterns. This method scores 6.

Complexity (possibility of replication):

The complexity of a security label reflects the possibility of replication, with higher complexity indicating a lower possibility of replication. Conventional security labels based on a single deterministic technique, such as fluorescence (or phosphorescence) and structural color methods, score of 2. Responsive and fluorescence-structural color integrated methods can enhance the complexity of security labels to some extent but are still produced through deterministic processes, existing potential of counterfeiting, scoring of 4. In our work, the intrinsic randomness of nanoparticles during the evaporation process yields security labels with physically unclonable function (PUF) that is impossible to replicate, scoring 6.

Encoding capacity:

Security labels with low encoding capacity, even those generated through random processes, may be recreated using forceful methods. Single technique methods, such as fluorescence (or phosphorescence) and structural color, produce security labels with only a single level of encryption information with the weakest encoding capacity. In contrast, responsive and

fluorescence-structural color integrated methods offer dynamic or multilevel encryption information. Our work incorporates encryption information on both macroscopic and microscopic dimensions. The macroscopic dimension includes fluorescence information under UV light and reflective information under natural light. Additionally, microscopic PUF security labels have been proven to possess high encoding capacity through formula calculation. The multidimensional and multilevel encryption information provides the highest encoding capacity. The scoring for each method aligns with their complexity score.

Legibility:

Typically, there is a trade-off relationship between the legibility of encrypted information and the complexity (possibility of replication) in security labels. Fluorescence methods require UV light for reading encrypted information, providing high concealment and scoring of 4. Structural color methods allow naked-eye reading under natural light but lack concealment, scoring 6. Combining fluorescence and structural color leverages both advantages, offering readability and high concealment, scoring 6. In our approach, macroscopic structural color patterns offer primary encryption, UV-visible fluorescent patterns provide additional encryption, and microscopic PUF patterns add significant complexity, making the system difficult to decipher. This hierarchical encryption system, ranging from macro to micro and predictable to unpredictable, ensures high complexity and excellent legibility, scoring 6.

Table S1. A qualitative comparison of the different anti-counterfeiting techniques framed in terms of key requirements.

	Fluorescence (or phosphorescence)	Responsive fluorescence (or phosphorescence)	Structural color	Responsive structural	Fluorescence-Structural Color	Our work
Environmental durability	★☆☆	★☆☆	★★★	★★★	★★☆	★★★
Manufacturing simplicity	★★★	★★☆	★★★	★★☆	★☆☆	★★★
Complexity	★☆☆	★★☆	★☆☆	★★☆	★★☆	★★★
Encoding capacity	★☆☆	★★☆	★☆☆	★★☆	★★☆	★★★
Legibility	★★☆	★★☆	★★★	★★★	★★★	★★★
Ref.	1-3	4-7	8-11	12-15	16	

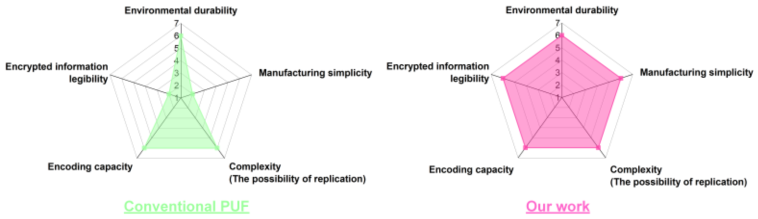

Figure S3. Radar plots of comparison schemes show the technological advances of our method relative to that of conventional PUF.

Optical PUF, based on intrinsic randomness and high encoding capacity, garnered extensive attention from other researchers, demonstrating the feasibility of its application in encryption and anti-counterfeiting. However, it has not been applied for practical application in encryption and anti-counterfeiting owing to a number of challenges such as the complex creation process and difficulty of determining signal extraction points. The novelty of our approach lies in overcoming these challenges by constructing a multiplex encryption system, ultimately harnessing the inherent randomness of PUFs to make the labels virtually impossible to replicate. Importantly, we present a more comprehensive solution, achieving a groundbreaking integration of encryption security and verification convenience. Macroscopic fluorescent and structural color patterns serve as easily identifiable entry points for encrypted information, while microscopic PUF security labels provide formidable encryption. This elucidates the advancement of our method compared to traditional PUFs.

Table S2. A qualitative comparison between the conventional PUF and our method is framed in terms of key requirements.

	Conventional PUF	Our work
Environmental durability	★★★	★★★
Manufacturing simplicity	★☆☆	★★★
Complexity	★★★	★★★
Encoding capacity	★★★	★★★
Legibility	★☆☆	★★★
Ref.	17-20	

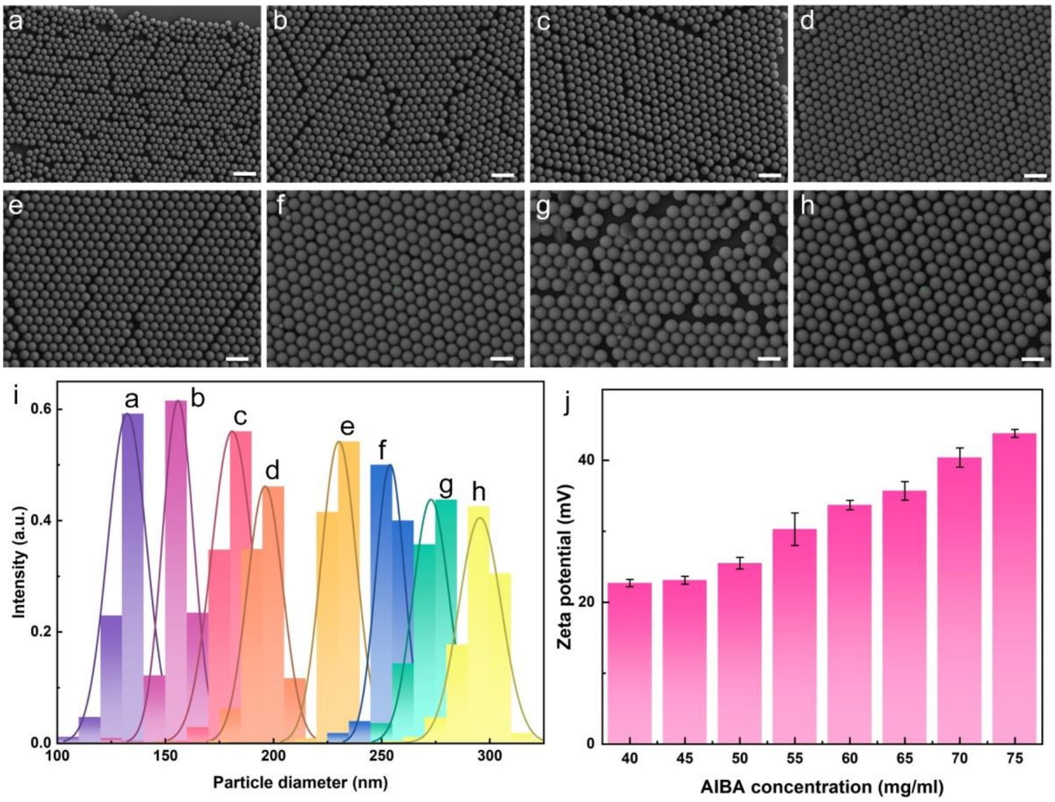

Figure S4. Synthesis of the monodispersed polystyrene (PS) nanoparticles using the microemulsion method: (a)–(h) SEM images of the synthesized monodispersed PS nanoparticles with different diameters (scale bar = 500 nm); (i) particle size distribution of the synthesized PS nanoparticles; (j) zeta potential values of the PS nanoparticles with various initiator concentrations.

The increase in initiator concentration (from 40 to 75 mg/mL) resulted in an augmented concentration of the polymer chains within the reaction system, which reduced the critical length necessary for microparticle formation. Consequently, a greater number of monomers was polymerized and attached to the surfaces of the nanoparticle cores. Within a specific range, a higher initiator concentration led to larger particle sizes of synthesized PS nanoparticles (Figure S2a–h and Figure S2i). Moreover, the increase in initiator concentration generated more free radicals during the reaction, resulting in higher zeta potentials of the synthesized PS nanoparticles (Figure S2j).

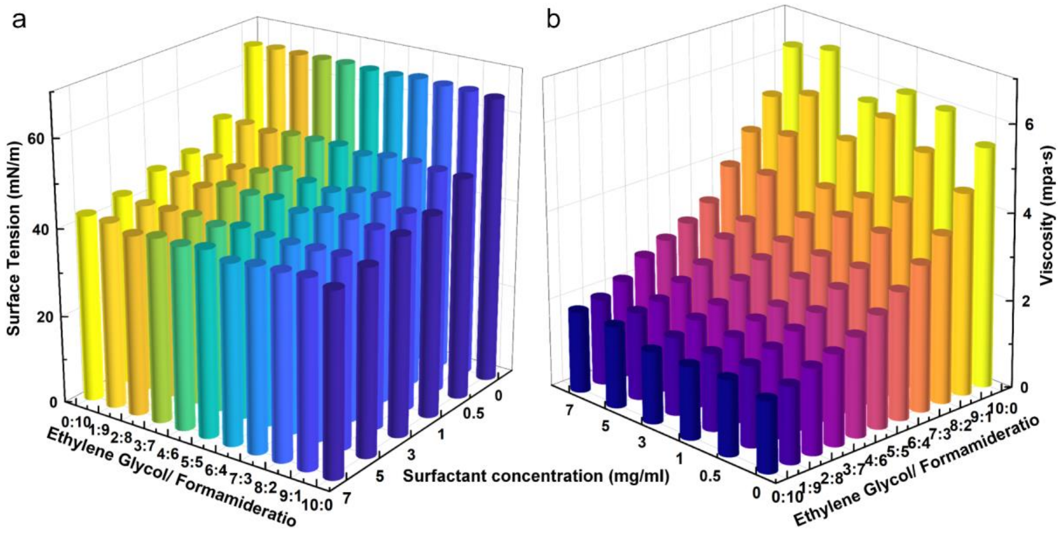

Figure S5. Variations of surface tension and viscosity of the colloidal photonic ink with various continuous phase compositions.

With an increase in surfactant (Brij-35) concentration, the surface tension of the ink gradually decreased from 65 to 41 mN/m, while the ratio of ethylene glycol (EG) to formamide (FA) showed a negligible effect on surface tension (Figure S3a). The viscosity of the inks within the system increased with increasing ethylene glycol ratio, ranging from 1.6 mPa·s with formamide and water as the sole solvent (EG:FA=0:10) to 6.3 mPa·s with ethylene glycol and water as the solvent (EG:FA=10:0). Similarly, the variations in surfactant concentration did not show a significant influence on the ink viscosity (Figure S3b).

In addition to ensuring ink printing stability, the binary solvent also induced Marangoni flow, which effectively prevented the formation of undesirable coffee rings during evaporation. This inward Marangoni flow also promoted the formation of ordered structures, while the presence of the surfactant contributed to Marangoni flow, facilitating the assembly of the nanoparticles and impeding crack formation during evaporation.

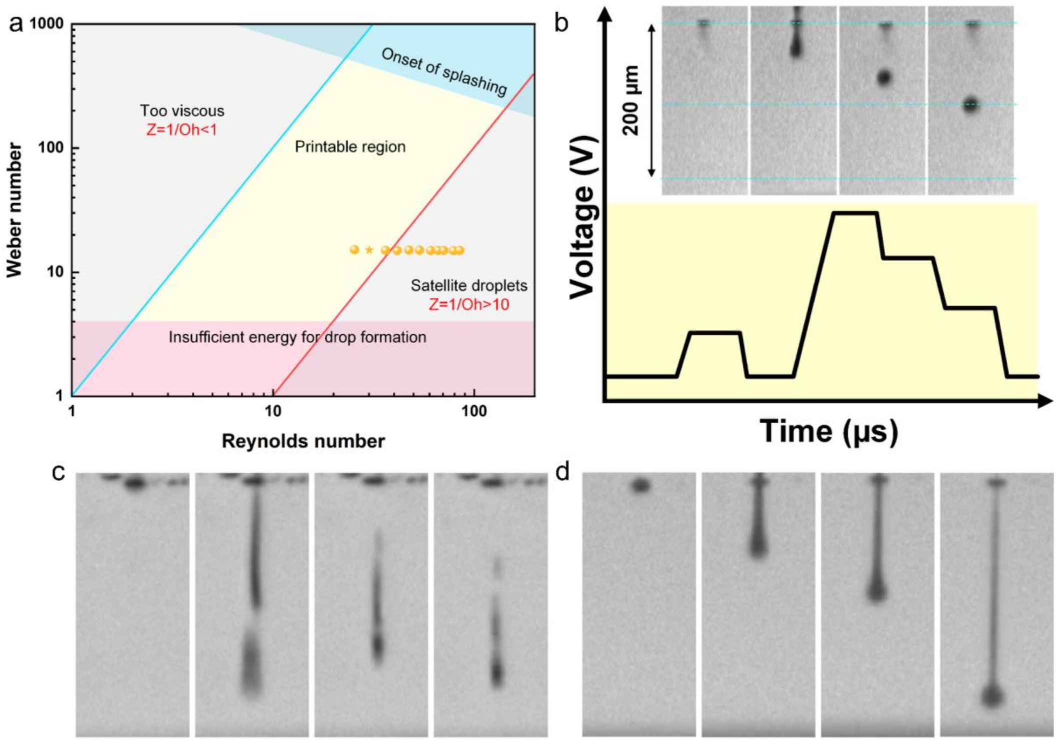

Figure S6. Fluidic characteristic parameters of the photonic ink with optimal continuous phase and voltage waveforms for inkjet printing: (a) positions of the fluidic characteristic parameters in the phase diagram for different glycol-to-formamide ratios, where the star-shaped points represent the optimal selected ink ratios (concentration of surfactant Brij-35: 0 mg/mL); (b) double pulse voltage waveform and corresponding ink drop flight status; (c) satellite dot; (d) trailing droplet.

The parameter *Re* represented the ratio of the inertial force to the viscous force in the fluid motion of the ink, while *We* denoted the ratio of the inertial force to the surface tension effect, and *Oh* combined these two parameters, describing the interplay between the viscous forces, inertial forces, and surface tension[21].

We used the reciprocal of *Oh*, *Z* (Equation S1) to quantify the printability of the ink, as follows:

$$Z = \frac{1}{Oh} = \frac{Re}{\sqrt{We}} \tag{S1}$$

When the value of *Z* was too low, the ink had a high viscosity, which impeded the ejection of droplets from the nozzle (left of the blue line in Figure S4a). Conversely, a higher value of *Z* resulted in the formation of numerous satellite droplets surrounding the main droplet during the printing process (right of the red line in Figure S4a), thus affecting the final print quality. Generally, droplet formation stabilized within the range of $1<Z<10$.

In addition, the influence of fluid/air surface tension at the nozzle served as another limiting factor in droplet generation, and the droplet had to contain sufficient energy to overcome this barrier before ejection. The minimum velocity (v_{min}) required for droplet ejection could be defined as:

$$v_{min} = \left(\frac{4\gamma}{\rho d_n} \right)^{1/2} \tag{S2}$$

where d_n is the diameter of the nozzle. Equation S2 could subsequently be reformulated in terms of *We*, to determine the minimum printable value (the red-shaded portion of Fig. S4a) as follows:

$$We = v_{min} \left(\frac{\rho d_n}{\lambda} \right)^{1/2} > 4 \tag{S3}$$

Finally, the collision impact between the ejected ink droplets and the substrate had to be taken into account. The droplet deposited on the substrate had to have adequate space for diffusion to prevent splattering (blue-shaded portion of Figure S4a). The threshold at which splattering occurred could be described by a dimensionless set of variables, combining *Re* and *We* as follows:

$$We^{1/2} Re^{1/2} > f(R) \tag{S4}$$

where *f(R)* is a function of surface roughness, and for flat and smooth surfaces, *f(R)*≈ 50.

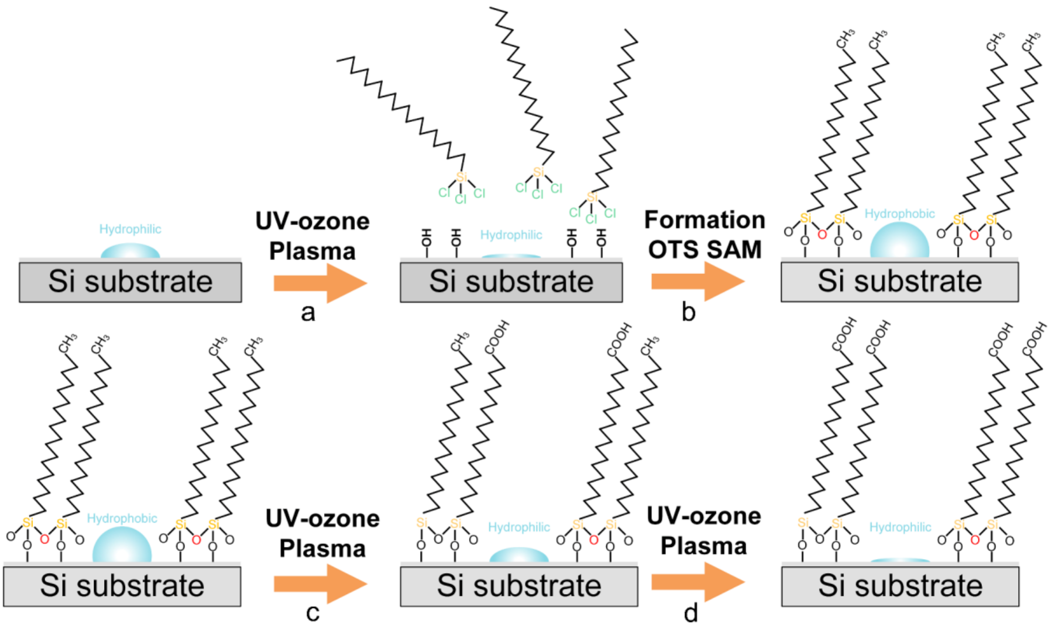

Figure S7. Schematic showing the hydrophobic and hydrophilic treatment process for the substrate.

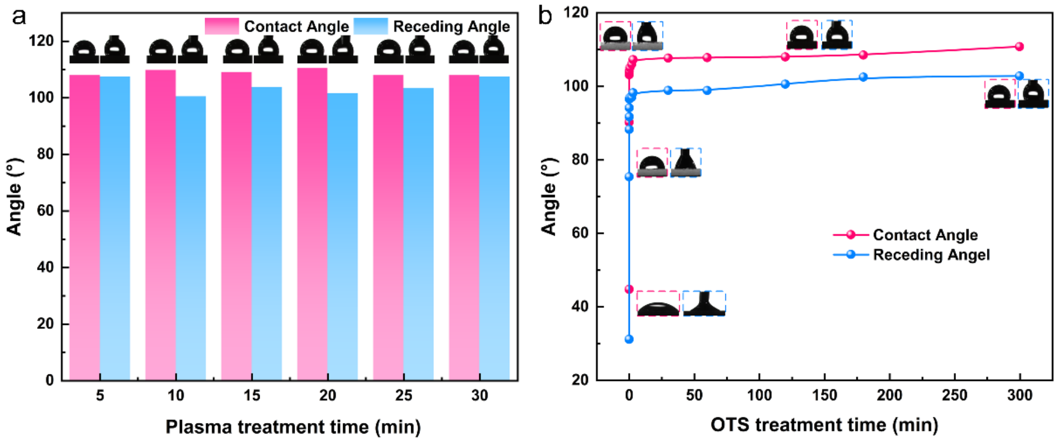

Figure S8. Variations in contact angle (CA) and receding angle (RA) during substrate hydrophobic treatment: (a) influence of UV-ozone plasma treatment duration prior to hydrophobic treatment on CA and RA for the final substrate; (b) variations in CA and RA with various OTS treatment time.

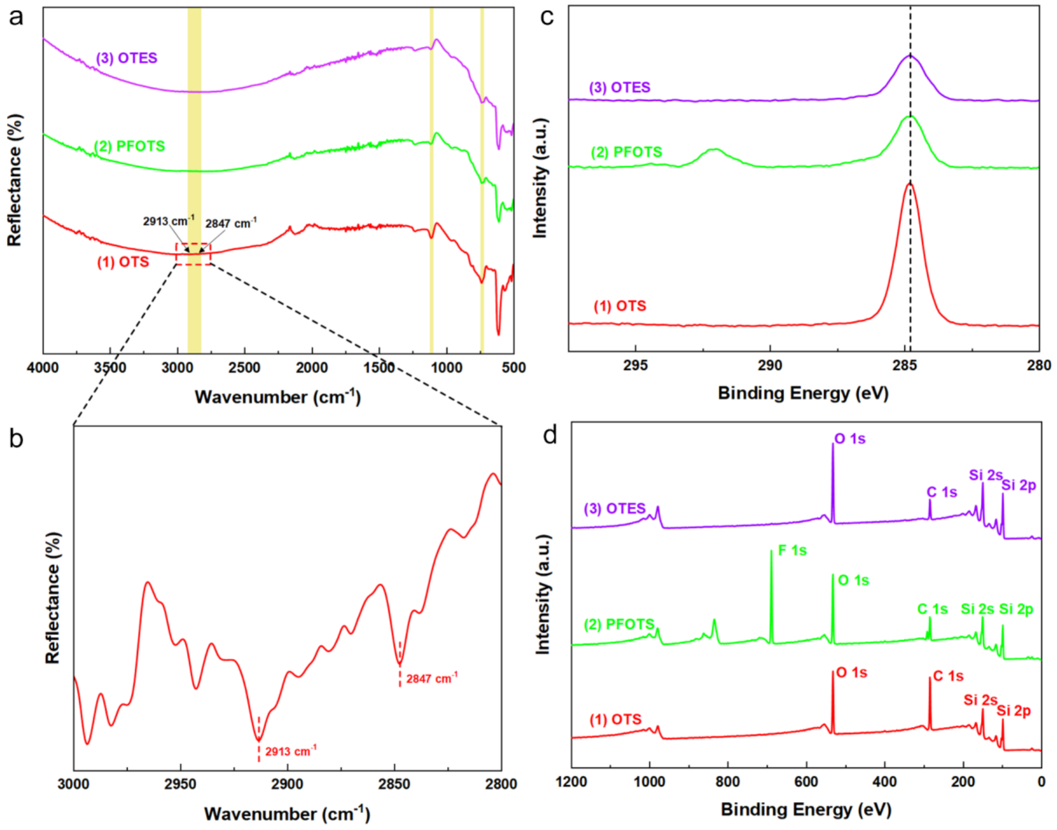

Figure S9. Composition characterization of the self-assembled monolayers obtained by hydrophobic treatment of the substrates with various silanes: (a) and (b) FT-IR spectra of the hydrophobically treated Si wafer; (c) and (d) XPS spectra of the hydrophobically-treated Si wafer.

Among the different silane reagents, the chlorosilane-treated substrate exhibited superior hydrophobicity compared to the ethoxysilane-treated substrate. This could be primarily attributed to the higher reactivity of chlorine compared to the ethoxyl groups, which had a larger molecular size, leading to a significant spatial barrier effect.

In the FTIR spectra, the peak at 738 cm^{-1} corresponded to the stretching vibration peak of Si-C, and the peak at 1112 cm^{-1} corresponded to the asymmetric stretching peak of Si-O-Si, which confirmed the formation of chemical bonding between the silane self-assembled monolayer (SAM) and the substrate (Figure S7a). Additionally, the peaks at 2847 cm^{-1} and 2913 cm^{-1} corresponded to the symmetric and asymmetric stretching vibrations of the long-chain alkyl -CH2- groups in OTS (Figure S7b). Figure S7c presents the C(1s) spectra of the SAMs that formed after treatment with different silane reagents. The substrate treated with OTS exhibited the highest intensity of the hydrocarbon chain (CH_x) peaks, which was consistent with the previously measured hydrophobicity of the substrate. The full XPS spectra (Figure S7d)

provided the atomic ratios of C/O/Si/F in the SAM formed by the different silane reagents, as follows: OTS (C 1s 41.35%, Si 2p 2.26%, O 1s 55.56%, and F 1s 0%); PFOTS (C 1s 8.47%, Si 2p 13.93%, O 1s 30.50%, and F 1s 46.46%); OTES (C 1s 13.56%, Si 2p 28.55%, O 1s 57.89%, and F 1s 0%).

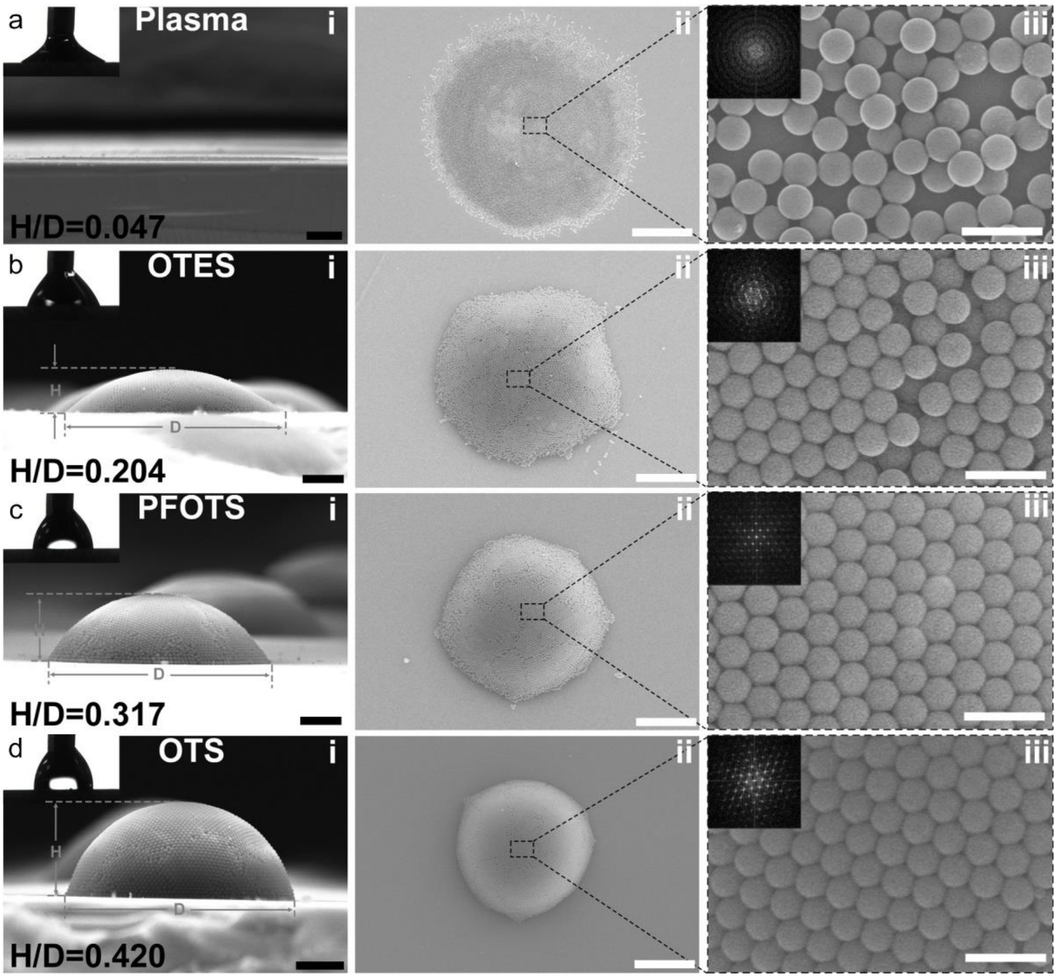

Figure S10. SEM morphology of the hemispherical colloidal nanoparticles aggregates formed through evaporation of the inkjet-printed droplets on different silane-treated substrates: (a) plasma treatment (hydrophilic); (b) OTES treatment; (c) PFOTS treatment; (d) OTS treatment; (i) side-view SEM images of the micro-domes formed by ink droplets drying on the substrates with different hydrophobicity characteristics (scale bar = 3 μm), where the insets show the corresponding receding angle image; (ii) top-view SEM images of the micro-domes (scale bar = 5 μm); (iii) partially enlarged top-view SEM images (scale bar = 500 nm), where the insets show the corresponding 2D fast Fourier transform (2D-FFT) images.

During evaporation of the colloidal photonic inks, islands of colloidal crystals formed on the curved surface of the droplet and exhibited different crystal orientations. In droplets with small RAs, large vacancies formed between the colloidal nanoparticles and released excessive strain energy. Therefore, the colloidal nanoparticles failed to assemble into colloidal photonic crystals and instead adopted a flattened shape with a small θ_{cua} between the colloidal aggregates and the substrate.

Conversely, when a significant contact radius (RA) existed between the droplet and the

substrate, the three-phase contact line could freely slide on the substrate, releasing stress among the colloidal nanoparticles. Moreover, grain boundaries consisting of tight 5–7 defects were introduced between the colloidal nanoparticles with distinct crystal orientations, thus alleviating the strain energy generated during assembly. Therefore, the colloidal nanoparticles could stack closely and form colloidal photonic crystals, and the θ_{cua} of the micro-domes with the substrate increased with increasing RA.

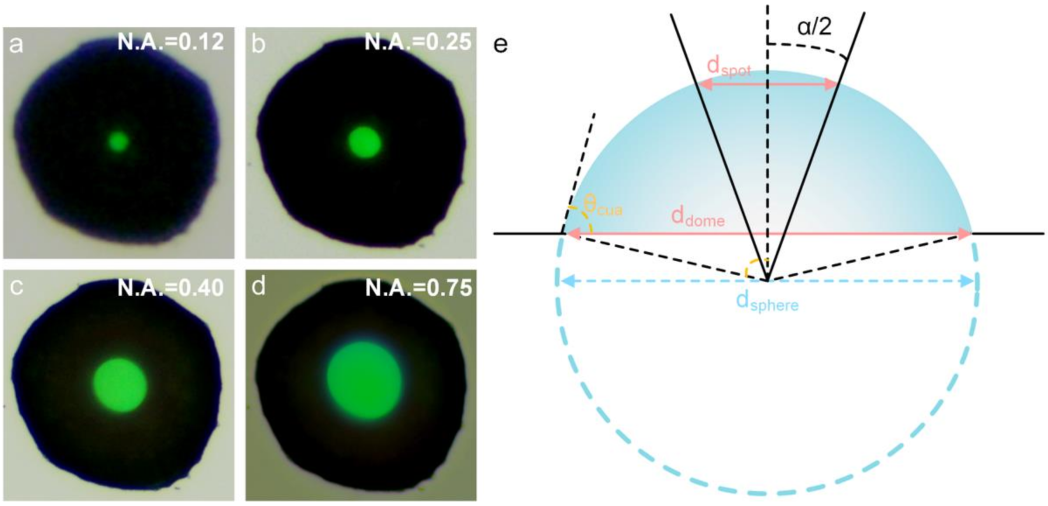

Figure S11. Optical details of a colloidal photonic crystal micro-dome observed under an optical bright field microscope: (a)–(d) colored spot in the central region of the micro-domes observed by objective lenses with different numerical apertures (NA); corresponding objective magnifications of 5×, 10×, 20× and 50×; (e) schematic showing the expected NA calculated from the ratio of colored spot to micro-dome diameters.

The radian angle corresponding to the colored spot in the center region of the micro-dome was the angle of incidence (α) of the incident light of the microscope. The numerical aperture of the microscope (NA) could be estimated by evaluating the ratio of the colored spot diameter (d_{spot}) to the micro-dome diameter (d_{dome})[22]:

$$\sin(d_{spot}/d_{sphere}) = \alpha/2 \tag{S5}$$

where d_{sphere} is the ideal sphere diameter. Due to the presence of a certain radian angle (θ_{cua}) between the micro-dome and the substrate, the relationship between the actual diameter of the micro-dome (d_{dome}) and the ideal sphere diameter (d_{sphere}) could be obtained by:

$$\sin(d_{dome}/d_{sphere}) = \theta_{cua} \tag{S6}$$

The numerical aperture (NA) of the microscope could be related to the aperture angle (α) and refractive index of the medium (n):

$$NA = n\sin(\alpha) \tag{S7}$$

The expected numerical aperture could be calculated by combining Equations S5–S7. The calculated microscope collection angles exhibited excellent agreement with the actual collection angles (Table S1).

Table S3. Comparison of the collection angle range of microscope lenses with different NA values and the actual observed color region.

Objective lens	5×	10×	20×	50×
NA	0.12	0.25	0.40	0.75
Actual collection angle (°)	6.9	14.5	23.6	48.6
d_{spot}/d_{dome}	0.09	0.14	0.22	0.43
Calculated collection angle (°)	10.2	16.8	25.9	50.2

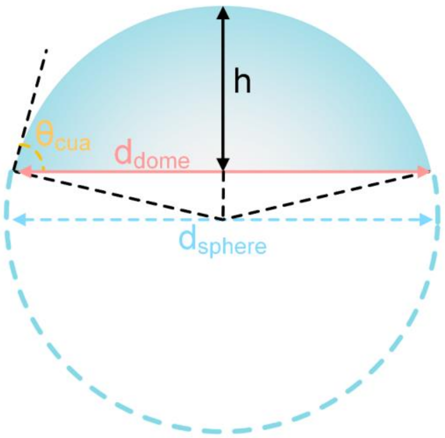

Figure S12. Mathematical geometric model showing the relationship of micro-dome volume with the diameter, height, curvature, and ideal sphere diameter.

The volume of a micro-dome (V_m) could be related to the diameter (d_{dome}), height (h), angle of curvature (θ_{cua}), and ideal sphere diameter (d_{sphere}), as follows[23]:

$$V_m = \pi \times h^2 (\frac{3}{2} d_{sphere} - h) / 3 \quad \text{(S8)}$$

According to the geometric relations, d_{sphere} and h could be replaced by d_{dome} and θ_{cua}:

$$d_{sphere} = d_{dome} \sin\theta_{cua} \quad \text{(S9)}$$

$$h = d_{dome}(1 - \cos\theta_{cua}) / 2\sin\theta_{cua} \quad \text{(S10)}$$

Thus, the volume of the micro-dome (V_m) could be re-expressed as:

$$V_m = \pi \times d^3 \frac{(2+\cos\theta_{cua})(1-\cos\theta_{cua})^2}{24(\sin\theta_{cua})^3} \quad \text{(S11)}$$

Setting $A = \frac{24(\sin\theta_{cua})^3}{(2+\cos\theta_{cua})(1-\cos\theta_{cua})^2}$, the micro-dome volume (V_m) could be replaced by the ink drop volume multiplied by the retention rate ($V_i \times R$), thus:

$$d = \sqrt[3]{V_i \times R \times A / \pi} \quad \text{(S12)}$$

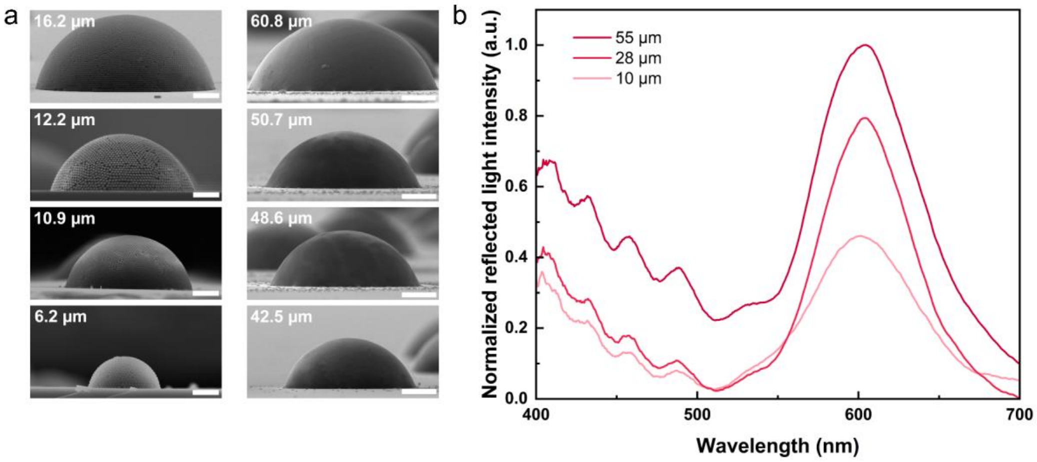

Figure S13. Morphology and optical properties of micro-domes with varying diameters: (a) representative side-view SEM images of micro-domes with different diameters (left: scale bar = 2 μm; right: scale bar = 10 μm); (b) representative reflectance spectra for different sized micro-domes.

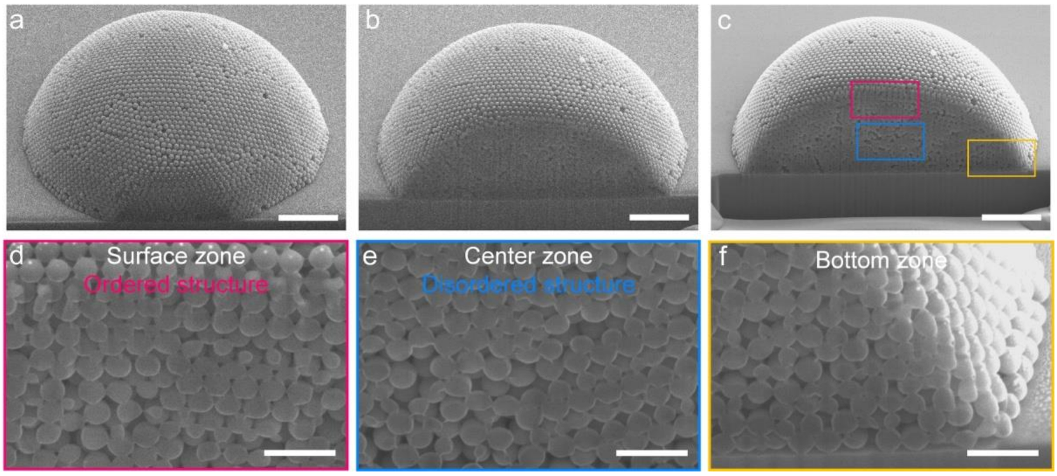

Figure S14. Focused ion beam (FIB) cutting colloidal photonic crystal, which assisted in observing the cross-section morphology of the micro-dome: (a)–(c) FIB cut through the colloidal photonic crystal micro-dome, showing the internal structure at various positions within the micro-dome (scale bar = 2 μm); (d) surface region of the micro-dome cross-section showing an ordered structure; (e) central region of the cross-section with a disordered structure; (f) bottom region of the cross-section; (d)–(f) (scale bar = 500 nm).

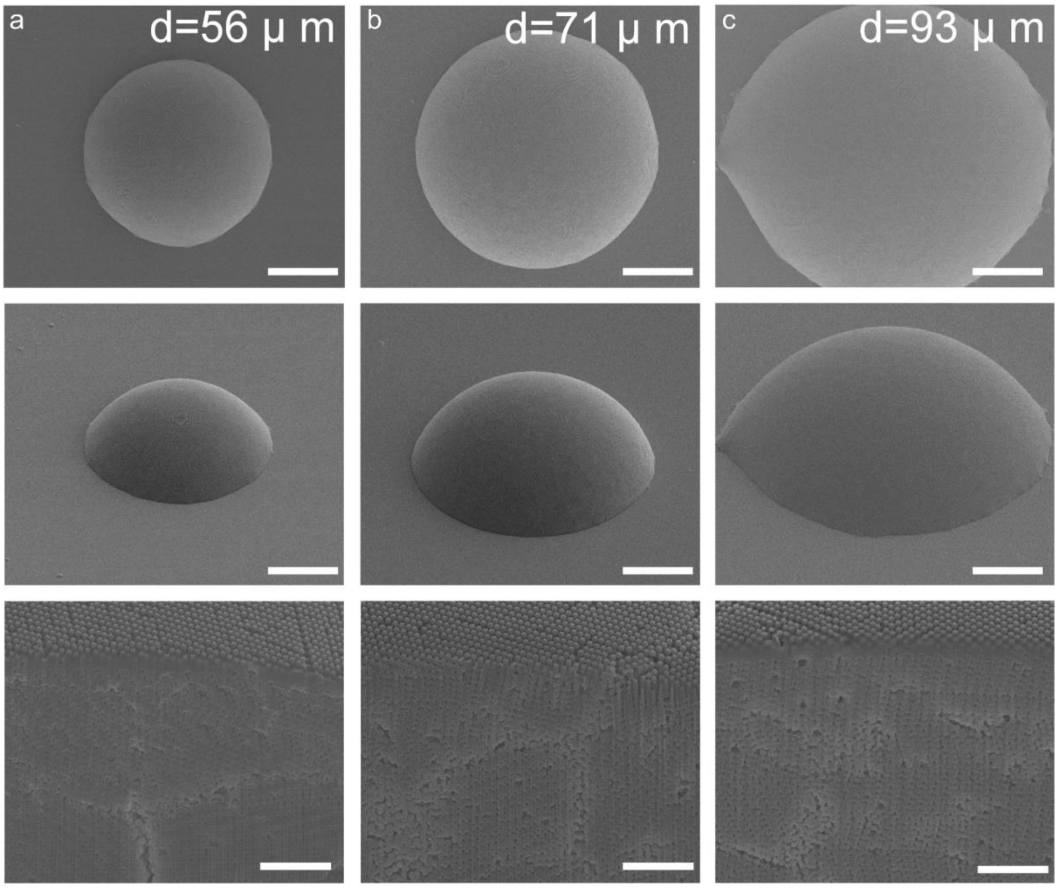

Figure S15. Top- and side-view images (scale bar = 20 μm) and FIB cross-section images (scale bar = 2 μm) of the colloidal photonic crystal micro-domes with different diameters.

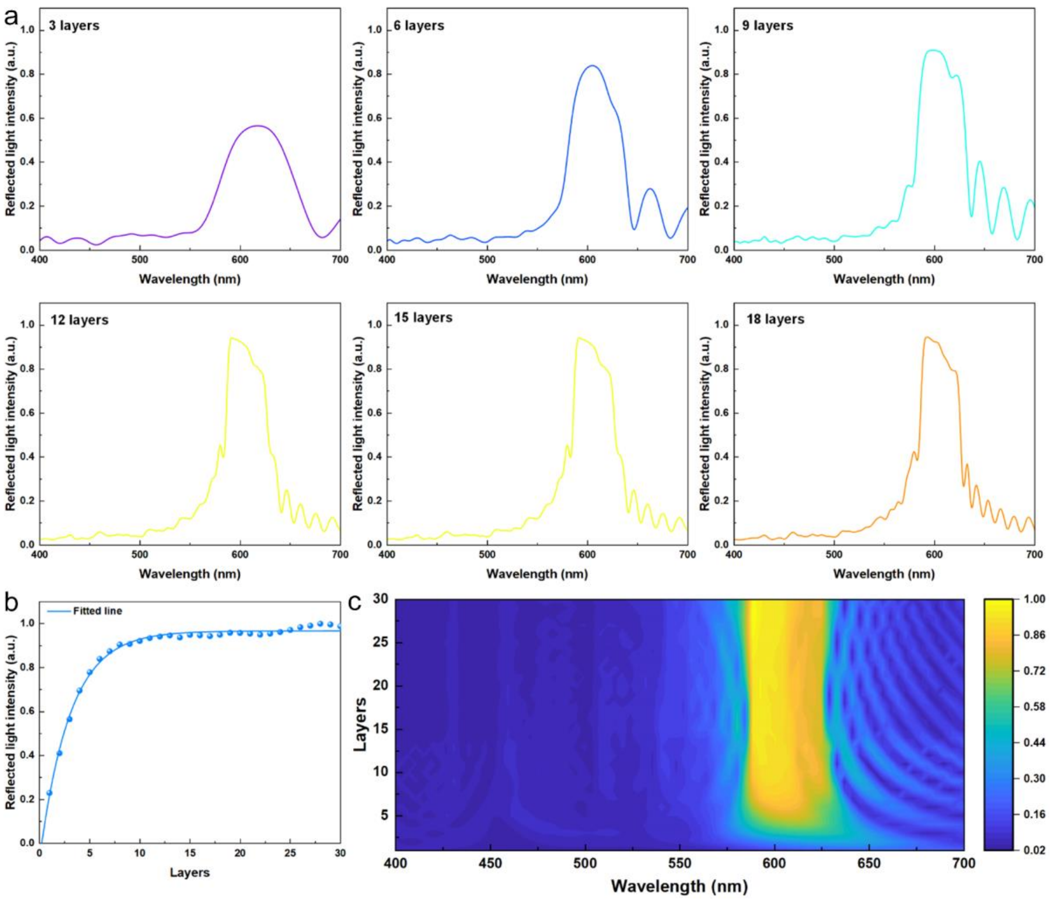

Figure S16. Numerical simulations used to calculate the reflectance spectra of the colloidal photonic crystal structures with different ordered layers: (a) numerical simulation used to calculate the reflection spectra, exemplarily shown for the multilayer structure composed of PS nanoparticles with a particle size of 255 nm and different numbers of ordered layers; (b) normalized peak reflection intensity for the different numbers of ordered layers; (c) numerical simulation of the 3D reflection spectra for the colloidal photonic crystals with different numbers of ordered layers.

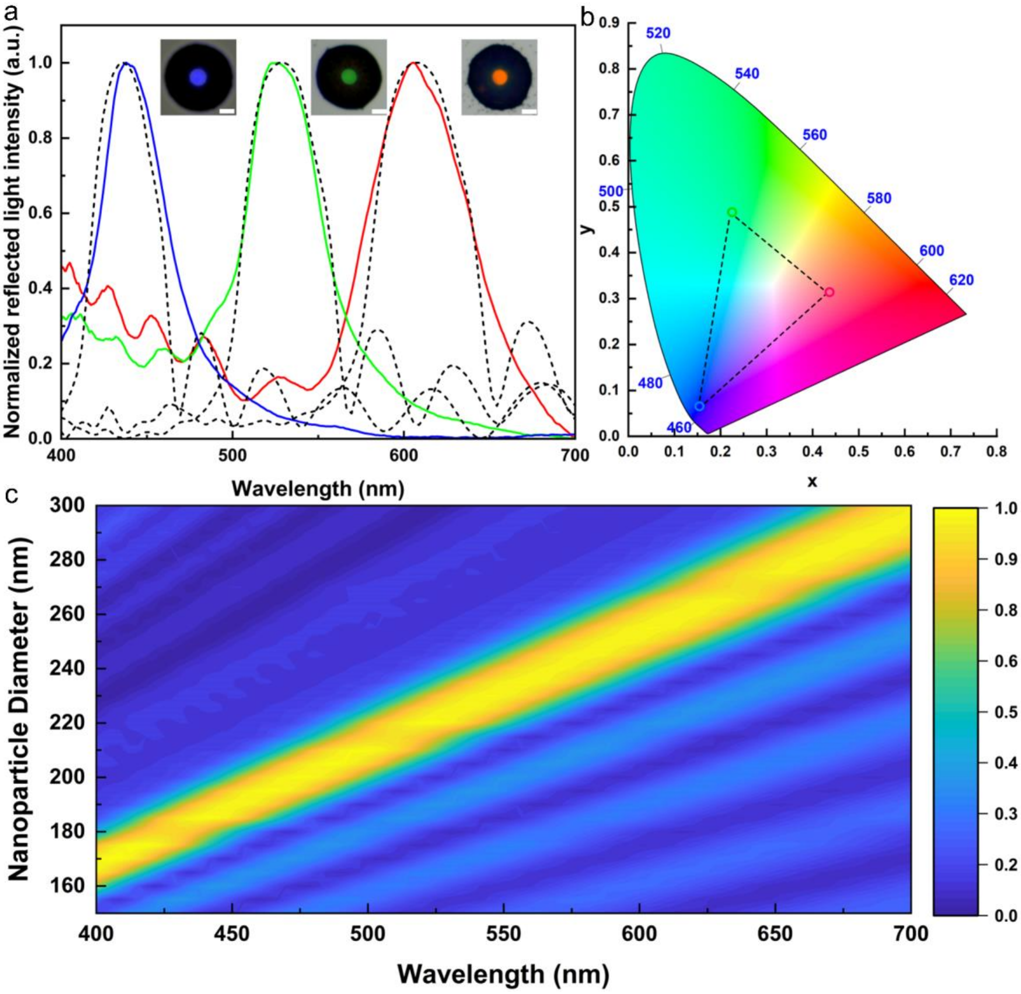

Figure S17. Particle size influence on Bragg diffraction for multichromatic structural colors: (a) reflection spectra of the colloidal photonic crystals (solid line) and simulated reflectance spectra of the PS nanoparticles based on the FCC crystal structure with (111) face stacking (dashed line); (b) commission Internationale de l'Éclairage (CIE) diagram illustrating the color coordinates of the colloidal photonic crystals with particle sizes of 180 nm (blue dots), 230 nm (green dots), and 254 nm (red dots); (c) numerical simulation of the 3D reflectance spectra of colloidal photonic crystal structural colors formed by self-assembled PS nanoparticles.

Table S4. Theoretical calculations and experimental measurements of the reflection peak wavelengths of the structural colors composed of colloidal nanoparticles with different particle sizes.

Particle size (nm)	Calculated peak wavelength (nm)	Measured peak wavelength (nm)
180	424	437
230	542	532
254	601	606

The peak wavelength of constructive interference in the colloidal photonic crystals was calculated by Bragg's law of diffraction, which is similar to crystallography[24]. By combining Snell's law of refraction with Bragg's law, the peak wavelength of a colloidal photonic crystal could be expressed by:

$$m\lambda = 2d\sqrt{n_{eff}^2 - \sin^2\theta} \tag{S13}$$

where d is the interplanar crystal spacing of the colloidal lattice, n_{eff} is the average refractive index of the colloidal photonic crystal, θ is the incidence angle of the incident light in the normal direction (perpendicular incidence of light θ = 0°), m indicates the reflection order, and λ is the reflection wavelength.

The colloidal nanoparticles were composed of closely stacked (111) faces of the face-centered cubic (FCC) structure, resulting in Bragg diffraction. The interplanar crystal spacing (d) could be calculated according to the particle size (D) by:

$$d = \sqrt{\frac{2}{3}}D \tag{S14}$$

The average refractive index (n_{eff}) of a colloidal photonic crystal could be calculated using the following equation:

$$n_{eff}^2 = n_a^2 f + n_b^2(1-f) \tag{S15}$$

where n_a and n_b denote the refractive indices of the two components constituting the colloidal photonic crystal, and in this study, consisted of polystyrene nanoparticles (n_a = 1.587) and air (n_b = 1), respectively, and f denotes the filling ratio of the colloidal particles (for the FCC structure, f = 0.74). Based on the above Equations (S13–S15), the peak reflection wavelengths for the colloidal photonic crystals could be calculated (Table S2), which coincided with the spectra measured by fiber optic spectroscopy.

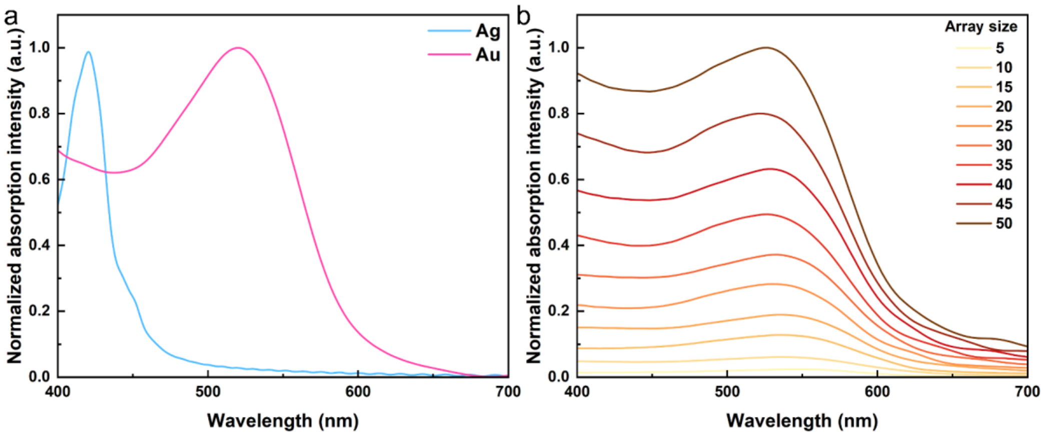

Figure S18. Selective absorption spectra of the noble metal nanoparticles (Au and Ag): (a) numerical simulations of the absorption spectra; (b) simulation of the representative number of Au nanoparticle array absorption spectra.

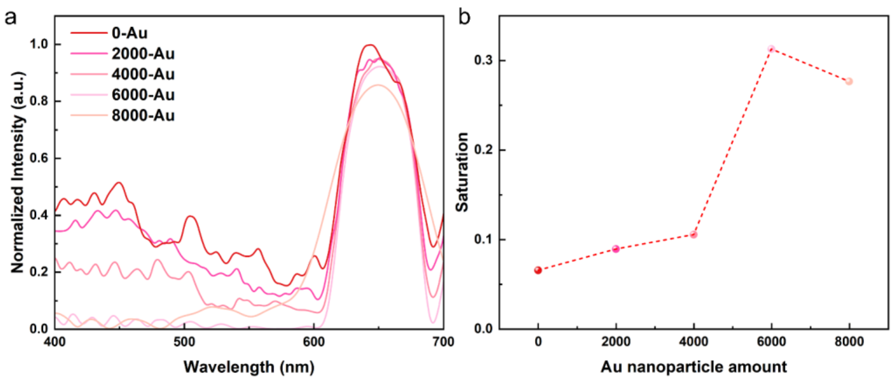

Figure S19. Effect of Au nanoparticle addition on the reflection spectra of colloidal photonic crystals, estimated by numerical simulations: (a) variations in Au nanoparticle content on the ordered and disordered colloidal nanoparticles structure; (b) dependence of the color saturation on the amount of Au nanoparticles added to the above structure.

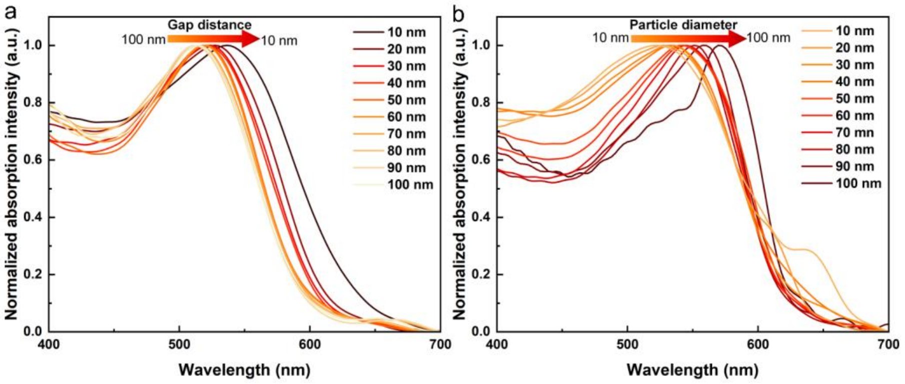

Figure S20. Dependence of Au nanoparticle simulation absorption spectra on the gap distance and nanoparticle diameter: (a) variations in Au nanoparticles array gap distance; (b) variations in Au nanoparticle diameter.

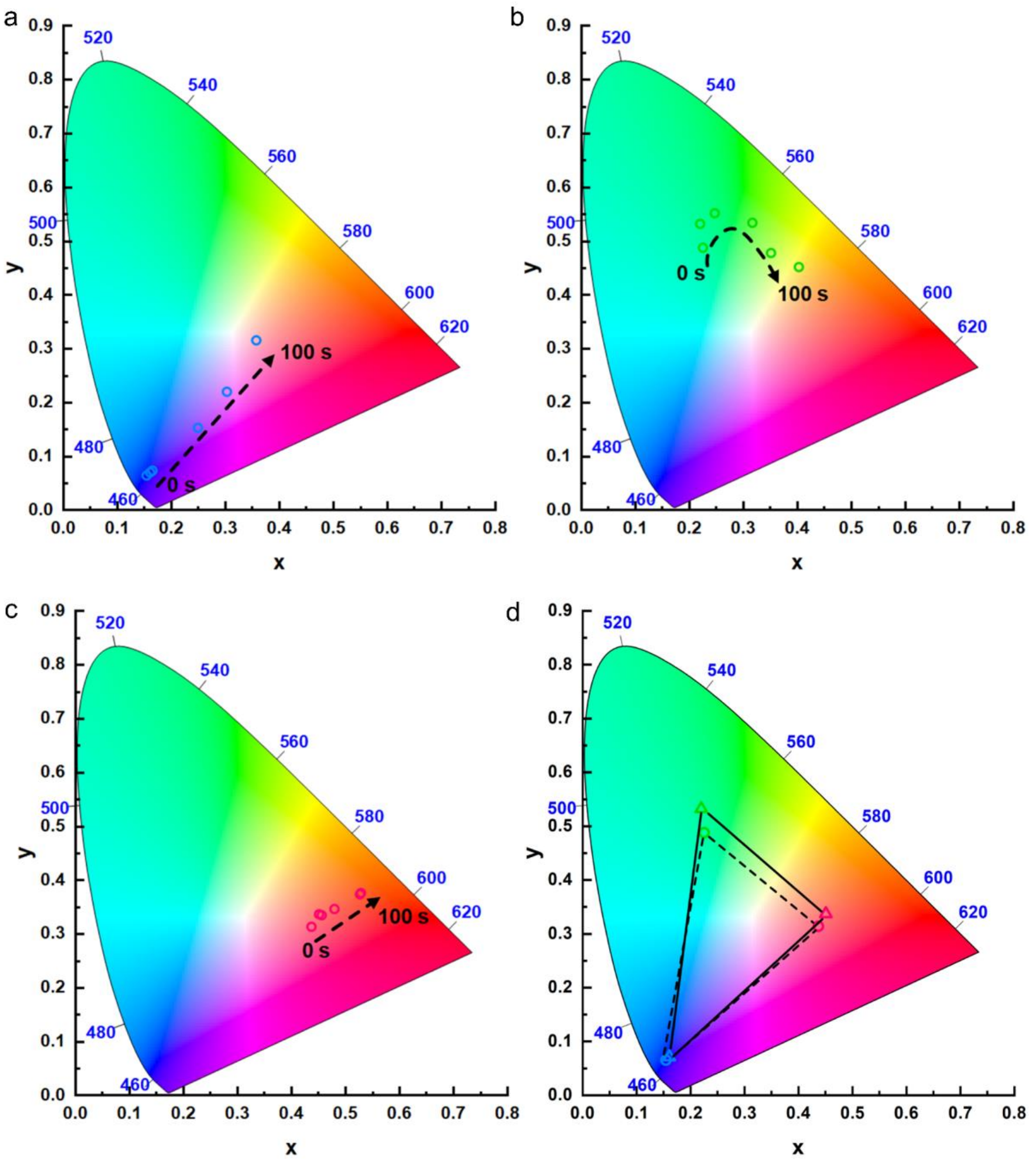

Figure S21. CIE diagram of the doped Au nanoparticles: particle sizes of (a) 180 nm; (b) 230 nm; (c) 254 nm; (d) change in color coordinates before (connected with solid lines) and after (connected with dash lines) doping with Au particles for 10 s.

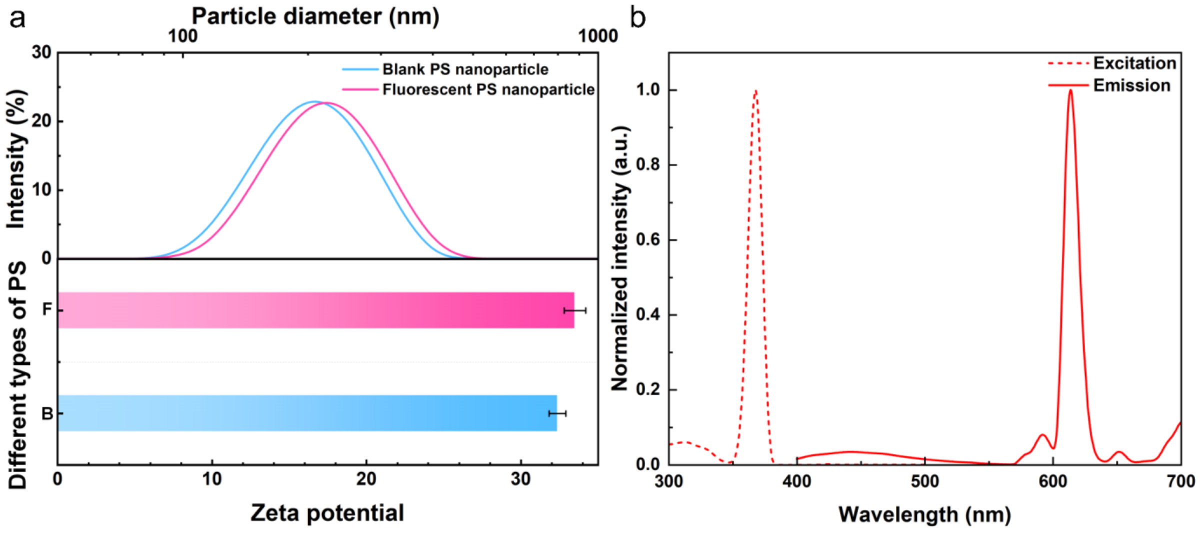

Figure S22. Comparison of the properties of fluorescent and blank PS nanoparticles: (a) particle size distribution and zeta potential; (b) fluorescence excitation and emission spectra.

Fluorescent PS nanoparticles were synthesized employing an Eu (III) complex as a fluorescent agent. Initially, $EuCl_3 \cdot 6H_2O$ was dissolved in 5 mL of ethanol, yielding a transparent solution. Under stirring, 8 mL of the ethanol-dissolved BFA solution was added. Subsequently, the resulting mixture was combined with a solution of TPPO dissolved in 5 mL of ethanol, and the $Eu(BFA)_3(TPPO)_2$ complex was obtained after a 6 h reaction at 50°C. Finally, the fluorescent agent, styrene, surfactant, and initiator were mixed, and we followed the same steps for synthesizing the blank PS nanoparticles to generate fluorescent PS nanoparticles. This method encapsulated the fluorophores within the microspheres, effectively preventing fluorescence quenching and mitigating the impact of environmental factors on fluorescence intensity. By adjusting the initiator concentration, we could obtain fluorescent and blank PS nanoparticles with similar particle sizes, which both exhibited Zeta potentials exceeding 30 mV for sufficient colloidal stability. The synthesized fluorescent PS nanoparticles had excitation and emission peak wavelengths of 368 nm and 614 nm, respectively.

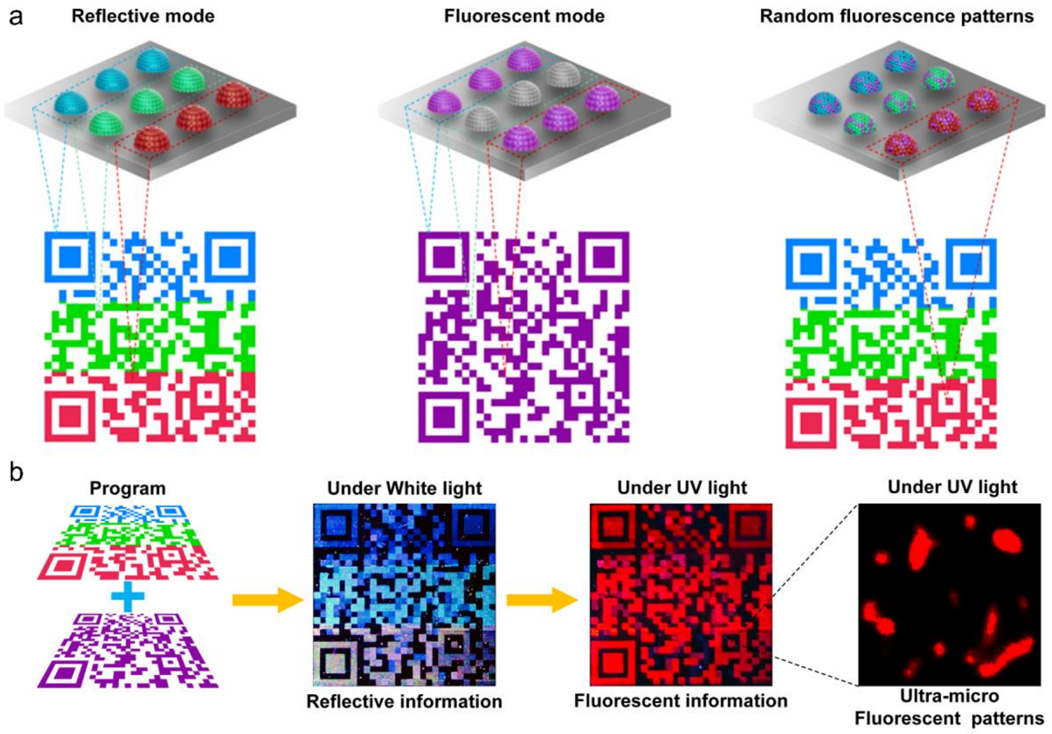

Figure S23. Fluorescence and structural color multiplex encryption system: (a) schematic showing the construction of multiplex encryption system; (b) security labels storing different encrypted information under reflective and fluorescent modes.

Initially, macroscopic anti-counterfeiting labels (Figure S21a and b) were designed to carry different information in reflective and fluorescent states, composed of colloidal nanoparticles with fluorescent or blank colloidal nanoparticles and varying particle sizes. For example, a blue structural color in the schematic was formed using 180 nm fluorescent PS nanoparticles, while the green structural color was composed of 230 nm blank PS nanoparticles. Subsequently, ink containing a mixture of fluorescent and blank colloidal nanoparticles of the same size (50% fluorescent particles and 50% blank colloidal particles) was applied at specific positions. PUF patterns were generated through the random distribution of nanoparticles during solvent evaporation.

Colloidal nanoparticles of different sizes formed colloidal photonic crystals, providing consumers with visible reflective information under white light. The aggregated assembly of fluorescent colloidal nanoparticles provided fluorescent information visible under UV light. The macroscopic structural color and fluorescent security labels realized dual-mode encryption in the visible and UV spectral regions, providing consumers with a lower recognition threshold.

In addition, the microscopic random fluorescent patterns could establish a deep learning-based database for physically unclonable encryption and anti-counterfeiting, which was difficult to reproduce or copy.

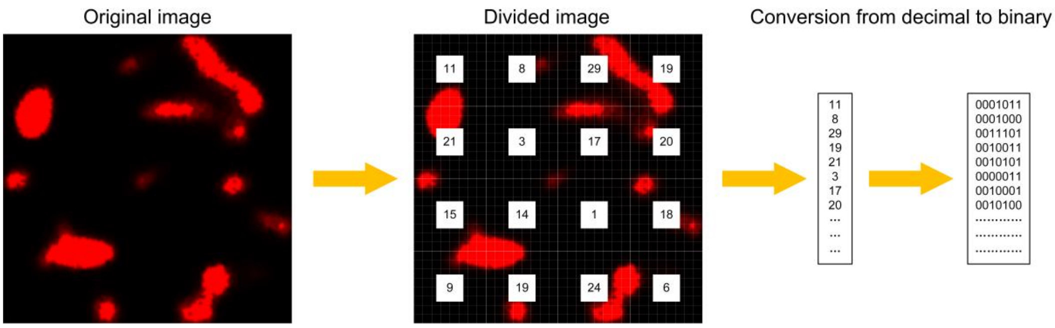

Figure S24. Conversion of fluorescence patterns to a binary format suitable for randomness tests.

The conversion of random fluorescent patterns into a binary format suitable for randomness testing involved several steps. First, the fluorescent pattern was divided into 16 (4×4) regions, each consisting of 64 (8×8) sub-regions. Subsequently, the number of red sub-regions in each region was counted in decimals, and this decimal number was converted into a 7-bit binary number. Combining all binary numbers from each region resulted in a random digital string generated by the PUF label. This method of obtaining a random digital string prevented the formation of 0 or 1 clusters formed by the direct conversion of fluorescent patterns.

Different statistical parameters (entropy, inter-hamming distance, and correlation coefficient) were employed to assess randomness. Entropy is a metric used to measure randomness or uncertainty, with higher entropy indicating greater randomness and lower entropy suggesting more regularity. The formulas and meanings for each parameter were as follows:

$$Entropy = -\sum_{i=1}^{n} P(x_i) \cdot \log_2(P(x_i)) \quad \text{(S16)}$$

where n represents the number of possible values for the random variable, which was 2 in this context, x_i denotes the possible values of the random variable, and $P(x_i)$ is the probability of the random variable taking the value x_i.

The Inter-Hamming distance quantified the dissimilarity between the two different sets, i.e., the number of differing elements at corresponding positions in two equally long strings.

$$HD = \frac{\#\ of\ different\ bits}{\#\ of\ total\ bits} \quad \text{(S17)}$$

The correlation coefficient measured the strength and direction of the linear relationship

between two variables. A value of 1 indicated a perfect positive correlation, −1 represented a perfect negative correlation, and 0 indicated no correlation.

$$CC = \frac{\sum (X - \overline{X})(Y - \overline{Y})}{\sqrt{\sum (X - \overline{X})^2}\sqrt{(Y - \overline{Y})^2}} \tag{S18}$$

where X and Y were two different sets of binary variables, while X_i and Y_i were elements in X and Y, and $\overline{X}$ and $\overline{Y}$ were the means of X and Y.

A random test suite (NIST) was also employed to further validate the randomness of the digital string obtained from the PUF[25]. Due to limitations in data volume, only 9 out of 15 tests were conducted. The p-values for each test were all greater than 0.01, indicating the passage of all randomness tests, where a p-value of 1 indicated that the digital string was complete randomness, while a p-value of 0 suggested apparent non-randomness.

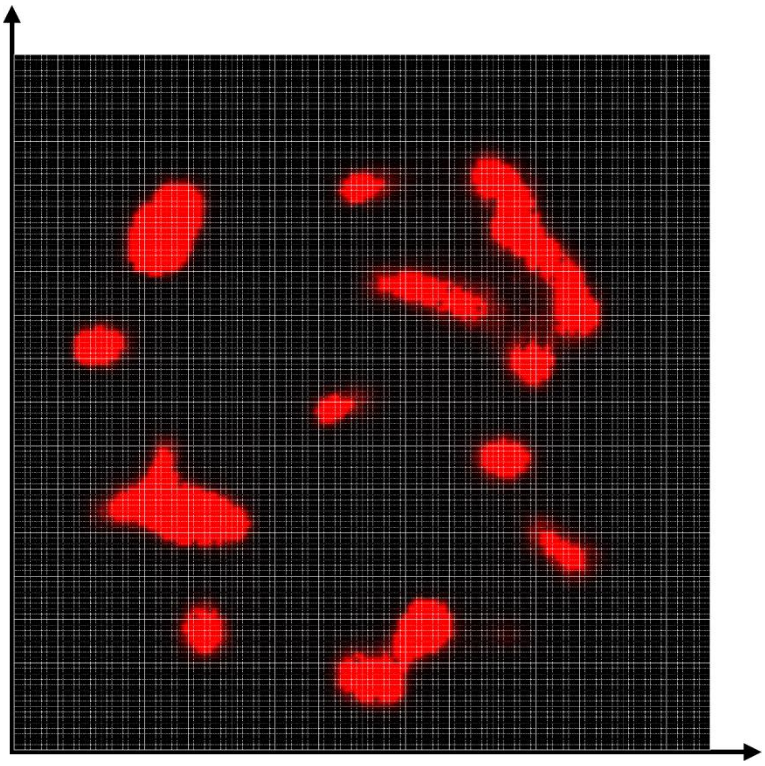

Figure S25. Encoding capacity estimation of a physically unclonable fluorescent pattern.

To estimate the encoding capacity, we defined the *X* and *Y* axes and divided the fluorescence pattern into 16 × 16 units, and each of these units was further divided into 8 × 8 subunits. Therefore, for a 512 × 512 pixel pattern, the subunit pixel length (*L*) was 4, and the resolution (*R*) was 128. We employed an image algorithm program to identify the pattern fill density (D), which was adjusted to 0.8 if it was below this threshold. We estimated the coding capacity using a *D* value of 0.8 as an example.

According to the estimation model developed by Migue et al., the encoding capacity (*#codesF*) of the fluorescence pattern (Figure S19) could be expressed by[26]:

$$\#codes = [C(1+L(\frac{1}{\sqrt{D}}\text{-}1))^2 + 1]^{\frac{D\times R^2}{L^2}} \tag{S19}$$

where C is the number of colors of a fluorescent pattern (1 in this study), based on Equation S16, the calculation showed that the encoding capacity of Figure S19 reached 1.35×10^{410}, which satisfied the requirement of not being decoded.

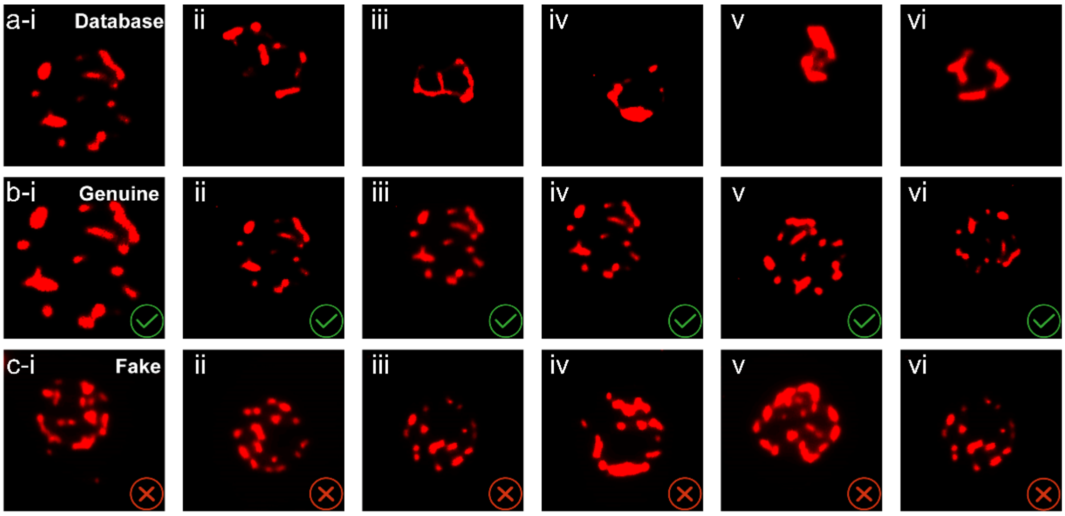

Figure S26. The generation of a database of PUF security labels: (a) microscopic fluorescence patterns (from genuine products) observed under a fluorescence microscope, which was used to establish a database for deep learning; (b) fluorescence patterns derived from the genuine product (a1) with different magnification, resolution, brightness, and rotation angle conditions, and combinations of these factors, forming datasets for training (80%) and authentication (20%); (c) fluorescent patterns not derived from the database (from fake products).

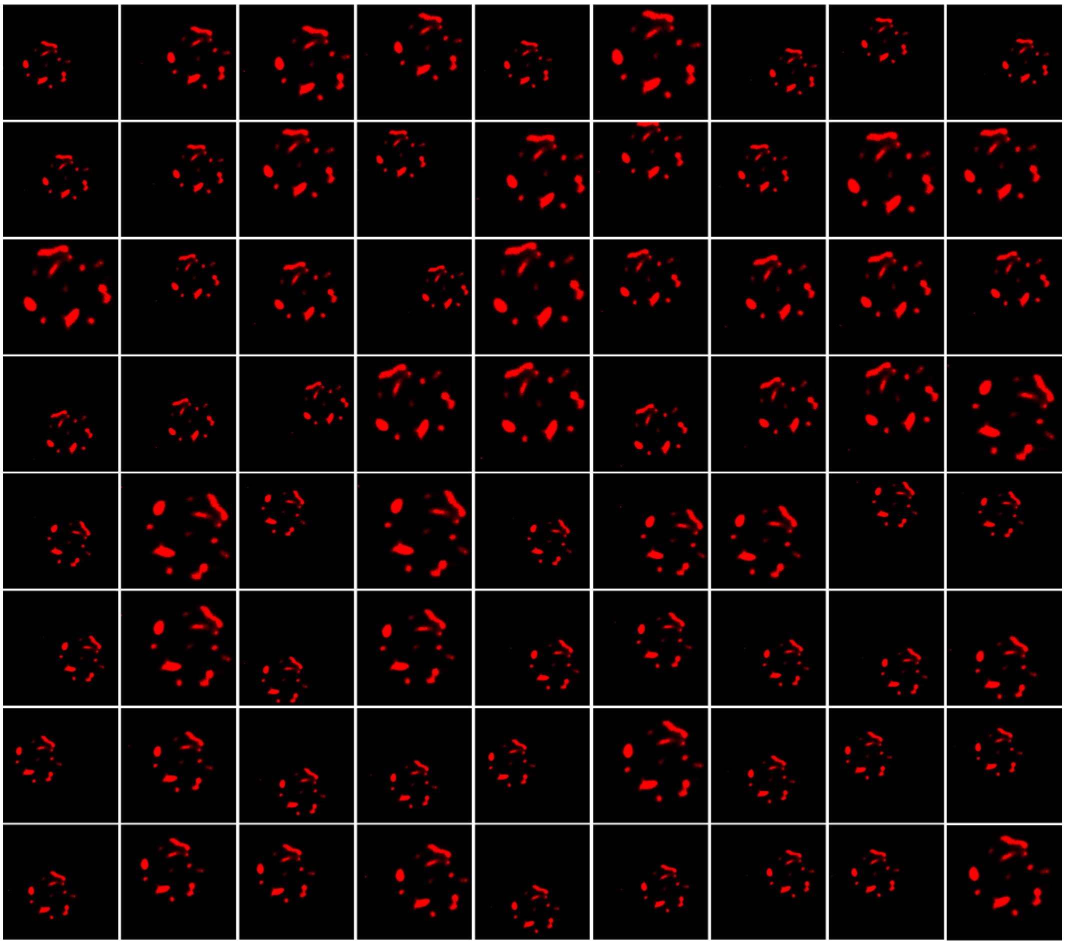

Figure S27. Selected 72 representative images used for deep learning.

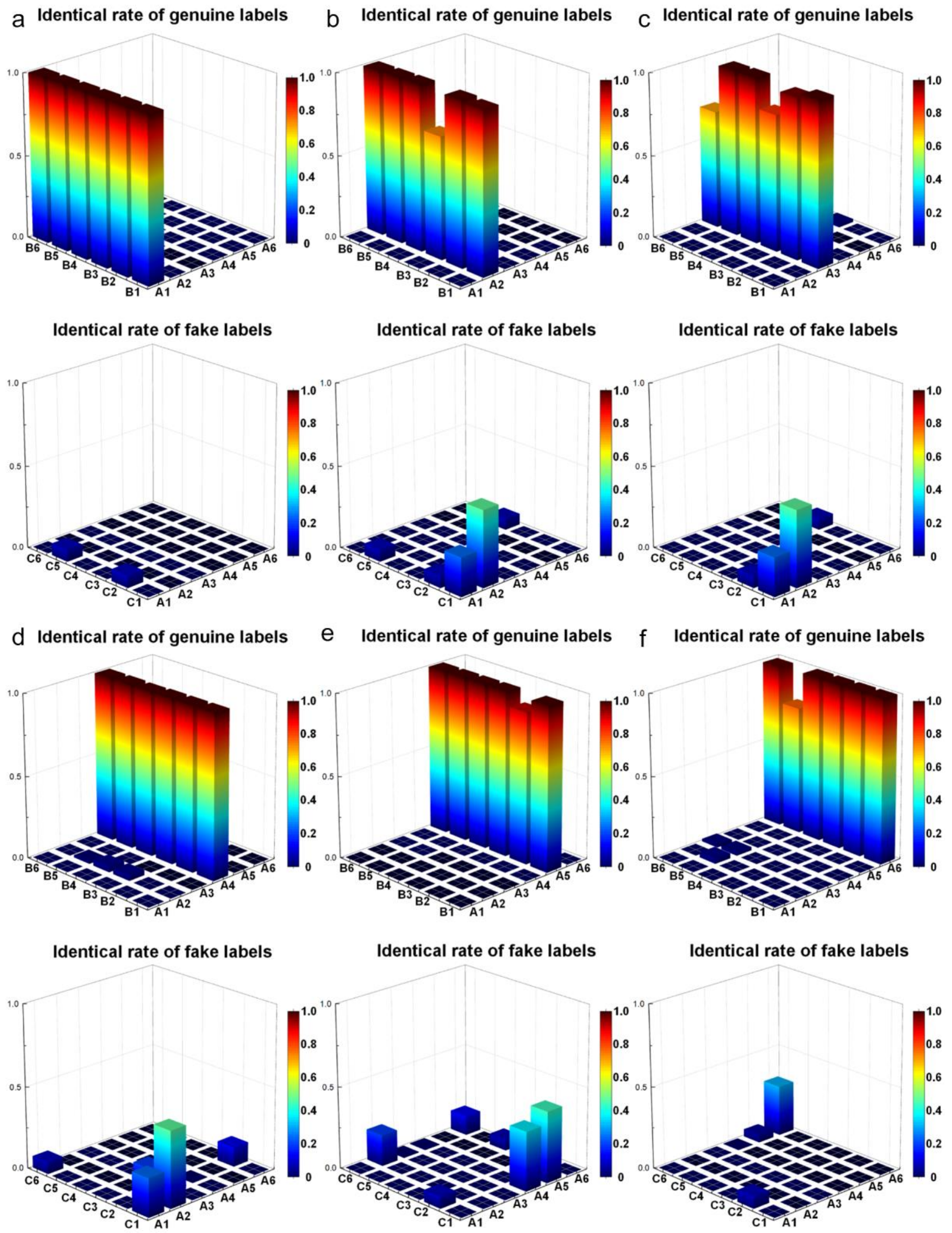

Figure S28. Matching degree for authenticity verification of PUF labels by the deep learning engine: fluorescent patterns from the database (genuine products, (a)–(f) top images) and fluorescent patterns not from the database (fake products, (a)–(f) bottom images).

Supplementary references

[1] M. Wang, K. Jiang, Y. Gao, Y. Liu, Z. Zhang, W. Zhao, H. Ji, T. Zheng, H. Feng, *Nanoscale* **2021**, *13*, 14337.

[2] K. Chang, Z. Liu, H. Chen, L. Sheng, S. X. A. Zhang, D. T. Chiu, S. Yin, C. Wu, W. Qin, *Small* **2014**, *10*, 4270.

[3] H. Chen, M. Wei, Y. He, J. Abed, S. Teale, E. H. Sargent, Z. Yang, *Nat. Commun.* **2022**, *13*, 4438.

[4] W. Tsai, Y. Lai, P. Tseng, C. Liao, Y. Chan, *ACS Appl. Mater. Interfaces* **2017**, *9*, 30918.

[5] S. Tian, H. Zhang, X. Yang, L. Yang, Q. Min, H. Ma, X. Yu, J. Qiu, X. Xu, *Chem. Eng. J.* **2021**, *412*, 128695.

[6] W. Huang, M. Xu, J. Liu, J. Wang, Y. Zhu, J. Liu, H. Rong, J. Zhang, *Adv. Funct. Mater.* **2019**, *29*.

[7] D. H. Park, W. Jeong, M. Seo, B. J. Park, J. M. Kim, *Adv. Funct. Mater.* **2016**, *26*, 498.

[8] H. Jiang, B. Kaminska, *ACS Nano* **2018**, *12*, 3112.

[9] M. M. Ito, A. H. Gibbons, D. Qin, D. Yamamoto, H. Jiang, D. Yamaguchi, K. Tanaka, E. Sivaniah, *Nature* **2019**, *570*, 363.

[10] M. Schwartz, G. Lenzini, Y. Geng, P. B. Rønne, P. Y. A. Ryan, J. P. F. Lagerwall, *Adv. Mater.* **2018**, *30*.

[11] K. Li, T. Li, T. Zhang, H. Li, A. Li, Z. Li, X. Lai, X. Hou, Y. Wang, L. Shi, M. Li, Y. Song, *Sci. Adv.* **2021**, *7*, eabh1992.

[12] L. Bai, Z. Xie, W. Wang, C. Yuan, Y. Zhao, Z. Mu, Q. Zhong, Z. Gu, *ACS Nano* **2014**, *8*, 11094.

[13] J. Chen, L. Xu, M. Yang, X. Chen, X. Chen, W. Hong, *Chem. Mater.* **2019**, *31*, 8918.

[14] J. Zhou, R. Chen, J. Wu, Z. Tang, G. Pan, Z. Fang, Y. Zhu, W. Lin, X. Lin, G. Yi, *ACS Appl. Mater. Interfaces* **2023**.

[15] X. Lin, D. Shi, G. Yi, D. Yu, *Responsive Mater.* **2024**, *2*.

[16] L. Qin, X. Liu, K. He, G. Yu, H. Yuan, M. Xu, F. Li, Y. Yu, *Nat. Commun.* **2021**, *12*, 699.

[17] J. Yang, M. Feng, J. Wang, Z. Zhao, R. Xu, Z. Chen, K. Zhang, A. Khan, Y. Han, F. Song, W. Huang, *Adv. Mater.* **2023**, *35*.

[18] Y. Li, Y. Mao, J. Wang, Z. Liu, P. Jia, N. Wu, H. Yu, J. Wang, Y. Song, J. Zhou, *Nanoscale* **2022**, *14*, 8833.

[19] Y. Liu, F. Han, F. Li, Y. Zhao, M. Chen, Z. Xu, X. Zheng, H. Hu, J. Yao, T. Guo, W. Lin, Y. Zheng, B. You, P. Liu, Y. Li, L. Qian, *Nat. Commun.* **2019**, *10*, 2409.

[20] Y. Li, Z. Liu, K. Zhu, L. Ai, P. Jia, N. Wu, H. Yu, J. Wang, X. Yao, J. Zhou, Y. Song, *Adv. Mater. Interfaces* **2021**, *8*.

[21] B. Derby, *Annu. Rev. Mater. Res.* **2010**, *40*, 395.

[22] N. Vogel, S. Utech, G. T. England, T. Shirman, K. R. Phillips, N. Koay, I. B. Burgess, M. Kolle, D. A. Weitz, J. Aizenberg, *Proc. Natl. Acad. Sci.* **2015**, *112*, 10845.

[23] K. Li, T. Li, T. Zhang, H. Li, A. Li, Z. Li, X. Lai, X. Hou, Y. Wang, L. Shi, M. Li, Y. Song, *Sci. Adv.* **2021**, *7*, eabh1992.

[24] R. Shanker, S. Sardar, S. Chen, S. Gamage, S. Rossi, M. P. Jonsson, *Nano Letters* **2020**, *20*, 7243.

[25] Andrew Rukhin, Juan Soto, James Nechvatal, Miles Smid, Elaine Barker, Stefan Leigh, Mark Levenson, Mark Vangel, David Banks, Alan Heckert, James Dray, San Vo, *National Institute of Standards & Technology* **2010**.

[26] M. R. Carro-Temboury, R. Arppe, T. Vosch, T. J. Sorensen, *Sci. Adv.* **2018**, *4*, e1701384.
